# Solar-Driven Redox Reactions with Metal Halide Perovskites Heterogeneous Structures

**DOI:** 10.1007/s40820-025-01886-y

**Published:** 2025-09-01

**Authors:** Qing Guo, Jin-Dan Zhang, Jian Li, Xiyuan Feng

**Affiliations:** 1 Xi’an Key Laboratory of Sustainable Energy Material Chemistry, Engineering Research Center of Energy Storage Materials and Devices, School of Chemistry, Xi’an Jiaotong University, Xi’an, 710049 People’s Republic of China; 2 Department of Physics, Zhejiang University of Technology, Hangzhou, 310023 People’s Republic of China; 3 Department of Physics, Gongshu Institute of Future Technology, ZJUT, Hangzhou, 310022 People’s Republic of China; 4 School of Microelectronics, Northwestern Polytechnical University, Xi’an, 710129 People’s Republic of China

**Keywords:** Metal halide perovskite, Heterojunction, Redox reaction, Solar-to-chemical conversion

## Abstract

Metal halide perovskites (MHPs) with striking electrical and optical properties have appeared at the forefront of semiconductor materials for photocatalytic redox reactions but still suffer from some intrinsic drawbacks such as inferior stability, severe charge-carrier recombination, and limited active sites. Heterojunctions have recently been widely constructed to improve light absorption, passivate surface for enhanced stability, and promote charge-carrier dynamics of MHPs. However, little attention has been paid to the review of MHPs-based heterojunctions for photocatalytic redox reactions. Here, recent advances of MHPs-based heterojunctions for photocatalytic redox reactions are highlighted. The structure, synthesis, and photophysical properties of MHPs-based heterojunctions are first introduced, including basic principles, categories (such as Schottky junction, type-I, type-II, Z-scheme, and S-scheme junction), and synthesis strategies. MHPs-based heterojunctions for photocatalytic redox reactions are then reviewed in four categories: H_2_ evolution, CO_2_ reduction, pollutant degradation, and organic synthesis. The challenges and prospects in solar-light-driven redox reactions with MHPs-based heterojunctions in the future are finally discussed.

## Highlights

- This paper reviews the fundamentals and research progress of metal halide perovskites (MHPs)-based heterojunctions for solar-driven redox reactions.
- A comprehensive summary is presented for the construction of various MHPs-based heterojunctions (e.g., Schottky-junction, type-I/II, Z-scheme, and S-scheme).
- The versatile use of MHPs-based heterojunctions in key photocatalytic redox reactions are summarized, including H_2_ evolution, CO_2_ reduction, pollutant degradation, and organic synthesis.

## Graphical abstract

**Figure:**
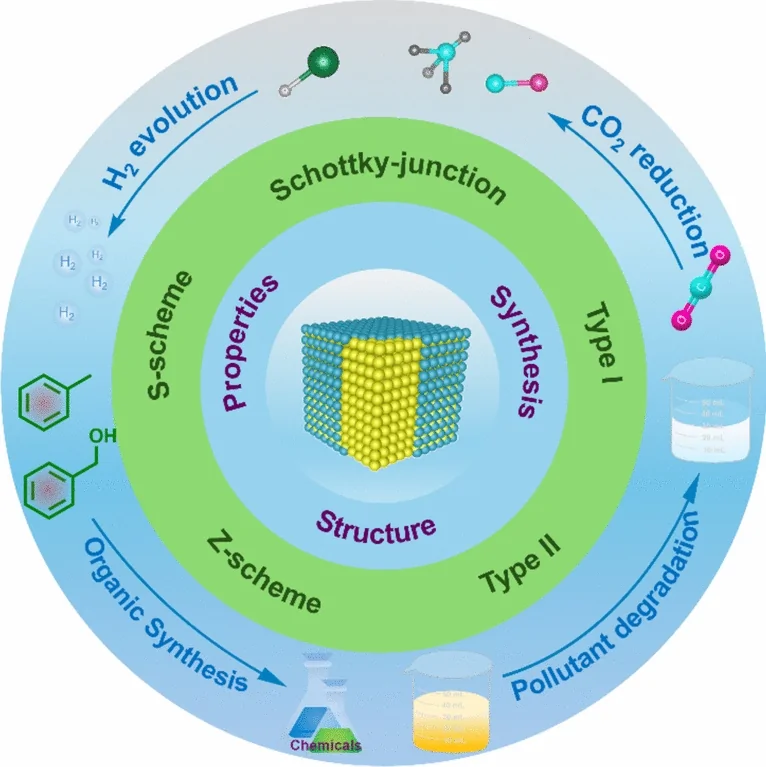

## Introduction

**Fig. 1:**
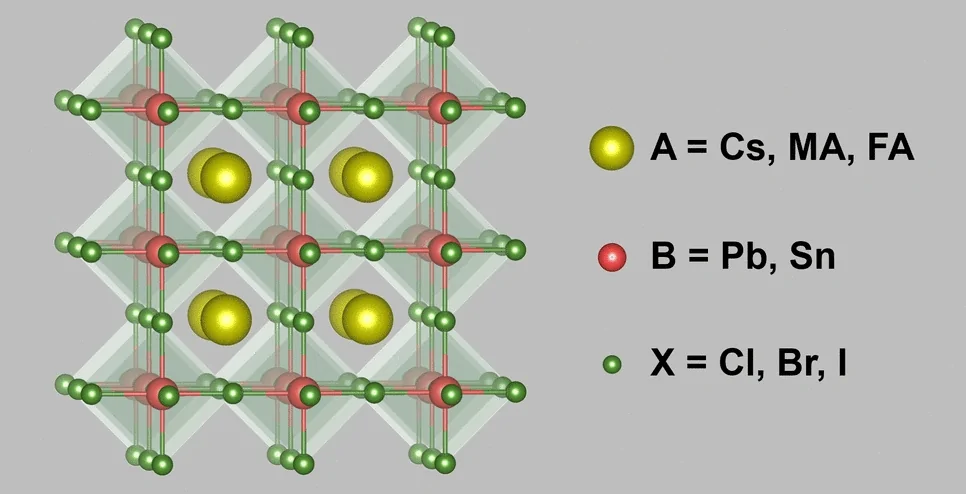
3D structure of MHPs

Owing to the advantages of mild reaction conditions and clean solar energy input, semiconductor photocatalysis technology exhibits great potential in resolving environmental concerns and energy crisis [1–5]. Semiconductor materials as the heart of photocatalytic technology have emerged in an endless stream. Metal halide perovskites (MHPs) with unique and outstanding optoelectronic properties have appeared at the forefront of semiconductor materials for photocatalysis [6]. The chemical formula of MHPs is ABX_3_, where A is a monovalent metal cation (e.g., Cs^+^) or an organic cation (e.g., CH_3_NH_3_^+^ (MA^+^), CH(NH_2_)_2_^+^ (FA^+^)), B is usually a divalent metal cation (e.g., Pb^2+^, Sn^2+^), and X is a halogen anion (e.g., Cl^−^, Br^−^, I^−^). The divalent metal B is surrounded by six halogen ions leading to a BX_6_ octahedral structure, while the A cation is in the cubo-octahedral cavity within the corner-shared BX_6_ octahedral framework forming a three-dimensional (3D) structure (Fig. 1) [7–9]. Compared to traditional II-VI, III-V, and IV-VI semiconductors, MHPs possess the unique advantages as following: i) the high molar extinction coefficient of MHPs (e.g., 10^5^–10^7^ L mol^−1^ cm^−1^ for CsPbX_3_ nanocrystals, ~ 10 times higher than that of CdSe with similar band gap) [10], is beneficial to the capture of solar light; ii) the optical band gaps could be facilely regulated for target reactions by quantum confinement effect or tuning compositions of anions. In combination with other striking electrical and optical properties, such as high carrier mobilities, long carrier-diffusion lengths, MHPs are considered as a promising class of candidate for photocatalytic redox reactions.

Inspired by the first photocatalytic work reported by Nam in 2016 [11], various MHP-based photocatalysts, such as CsPbX_3_ [12–14], Cs_3_Bi_2_X_9_ [15], CsSnX_3_ [16], and Cs_2_AgBiBr_6_ [17], have been explored for solar-light-driven reactions. However, the inferior stability, severe charge-carrier recombination, and limited active sites of bare MHPs greatly restrict the photocatalytic activity and durability. Many strategies have been developed to overcome these drawbacks, such as structural and compositional modifications [18–21]. For instance, dimensionality engineering of CsPbBr₃ significantly boosted H₂ evolution activity [22], while alkali metal doping—despite its non-active nature—unveiled the critical role of dopant sites in charge-carrier dynamics [23]. Furthermore, facet engineering enabled precise regulation of product yield and selectivity in photocatalytic organic synthesis [24], demonstrating the versatility of MHP photocatalysts design. Particularly, construction of heterojunction has shown great potential to optimize light absorption properties and photoinduced charge-carrier dynamics. The concept of heterojunction was first proposed in semiconductor physics by W. Shockley [25]. According to definition, heterojunctions are constructed by two or more semiconductors with similar crystal structures, coefficient of thermal expansions, and unequal band structure via physical or chemical bonding. Driven by built-in electric field at the interface, photogenerated electrons and holes could be spontaneously oriented and expedited transfer toward opposite direction, thus achieving much better photocatalytic performance than that of individual semiconductors [26]. To date, many MHPs heterojunctions have been developed and verified the effectiveness of heterojunction engineering on photocatalytic performance by enhancing light absorption ability, passivating surface, and promoting charge-carrier dynamics of MHPs. However, to the best of our knowledge, little attention has been paid to the summary of MHPs-based heterojunctions for photocatalytic redox reactions. A comprehensive and timely review concerning this field is highly desired, which will be significant for the design of future effective photocatalysts to realize solar-to-chemicals conversion.

**Fig. 2:**
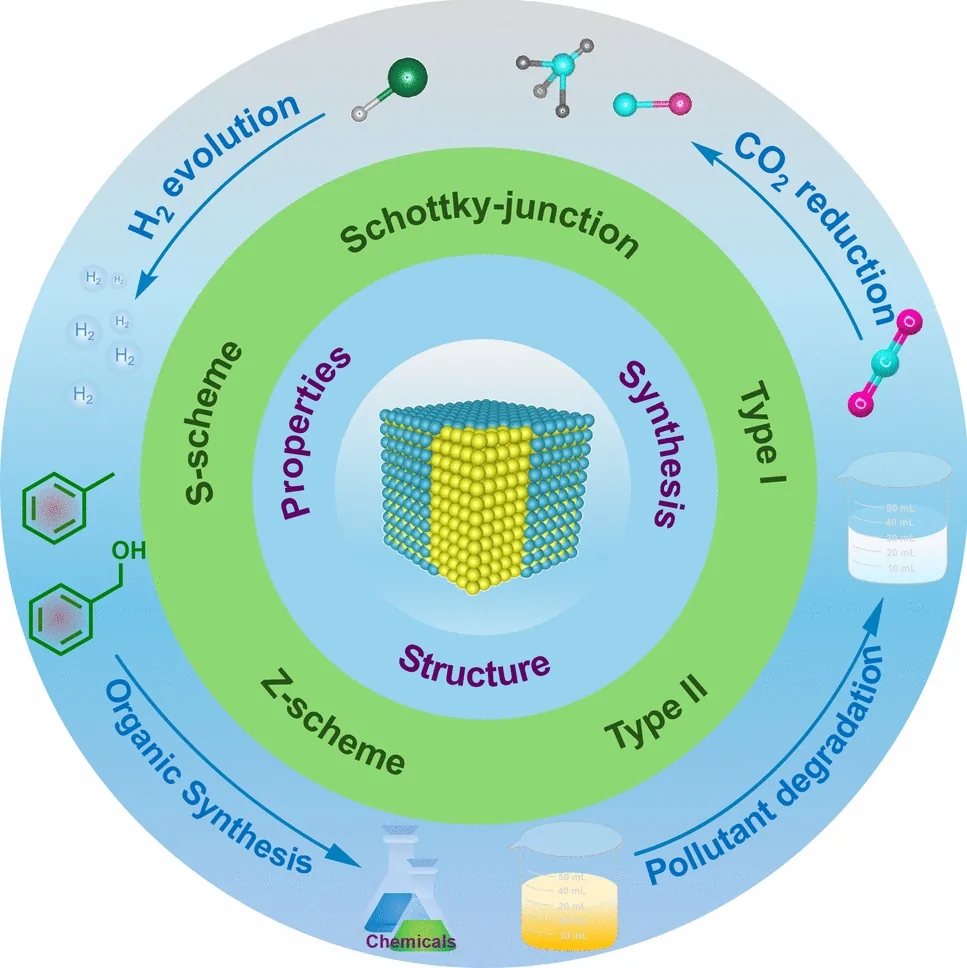
Schematic illustration of the full structure of this manuscript: type of heterojunction, the preparation methods, and the applicable reactions

This current review presents the recent progress of MHPs-based heterojunctions for photocatalytic redox reactions, as shown in Fig. 2. We first briefly introduce the structure, preparation methods, and photophysical properties of representative MHPs-based heterojunctions. Then, recent advances of MHPs-based heterojunctions in various photocatalytic redox reactions for improved photocatalytic performance and stability are introduced in terms of H_2_ evolution, CO_2_ reduction, pollutant degradation as well as organic synthesis. Finally, we present the possible challenges and prospects in this exciting field. This review gives the readers a clear picture about MHPs-based heterojunctions and provides guidance for designing advanced MHPs-based photocatalysts.

## Basic Principles, Categories, and Synthesis of MHPs-Based Heterojunctions

The structure of heterojunctions plays a key role in photocatalytic performance. In the following part, the basic principles, categories, and typical synthesis methods of MHPs-based heterojunctions will be outlined.

### Basic Principles of Heterojunction Photocatalysts

**Fig. 3:**
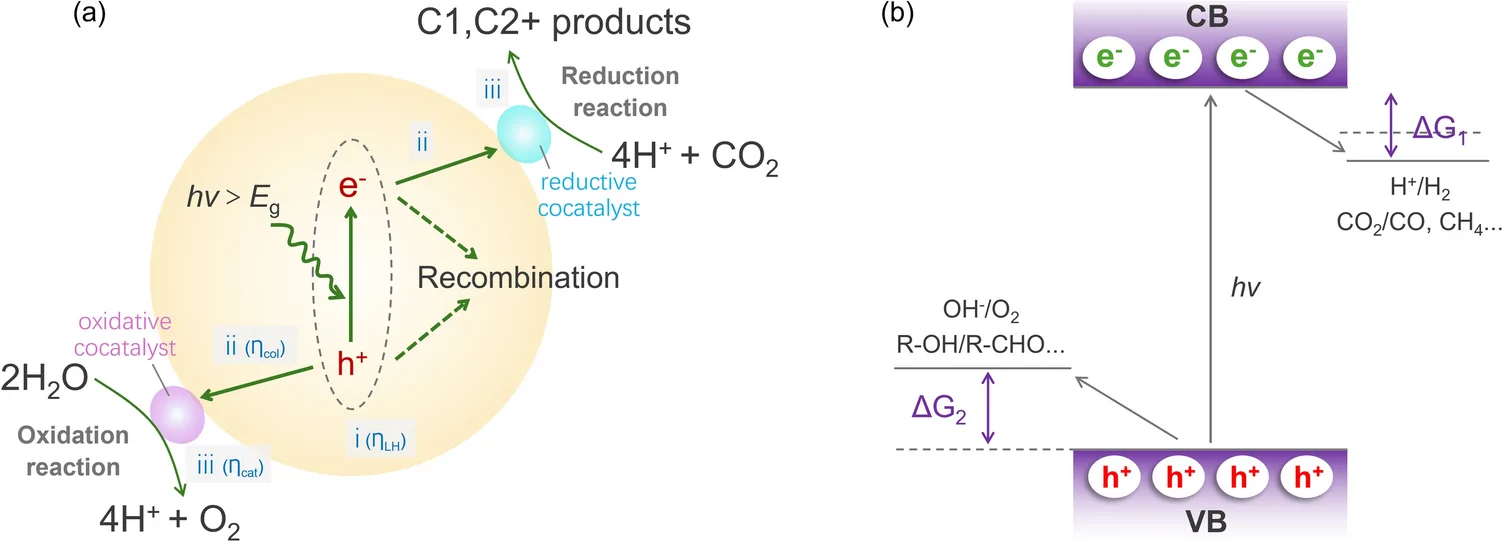
**a** General photocatalytic reaction process. **b** Energy diagram for photocatalytic reaction

In general, photocatalysis involves (Fig. 3a) [27–29]: (i) light absorption and generation of charge carriers (with the efficiency of η_LH_), in which the incident photon energy (*hv*) is equal to or larger than the band gap (Eg) of the semiconductors; (ii) separation of photogenerated charge carriers to produce electrons in the conduction band (CB) and holes in the valence band (VB) of semiconductors (with the efficiency of η_col_); (iii) redox reactions on active sites (with the efficiency of η_cat_) with electrons and holes in CB and VB as reductant and oxidant. To enable charge carriers to drive corresponding redox reactions, suitable band-position alignment of semiconductors should be considered to meet the thermodynamic requirements. The bottom of CB should be more negative than the reduction potential of electron acceptors (A, such as H^+^, CO_2,_ and so on), while the top of VB should be more positive than the oxidation potential of electron donors (D, such as sacrificial reagent, R-OH, and OH^−^) (Fig. 3b). However, photogenerated electrons and holes would be depleted with a seriously radiative recombination because of the extremely strong Columbic force between electrons and holes, and non-radiative annihilation at trap states in the form of emitted light and heat, respectively.

**Fig. 4:**
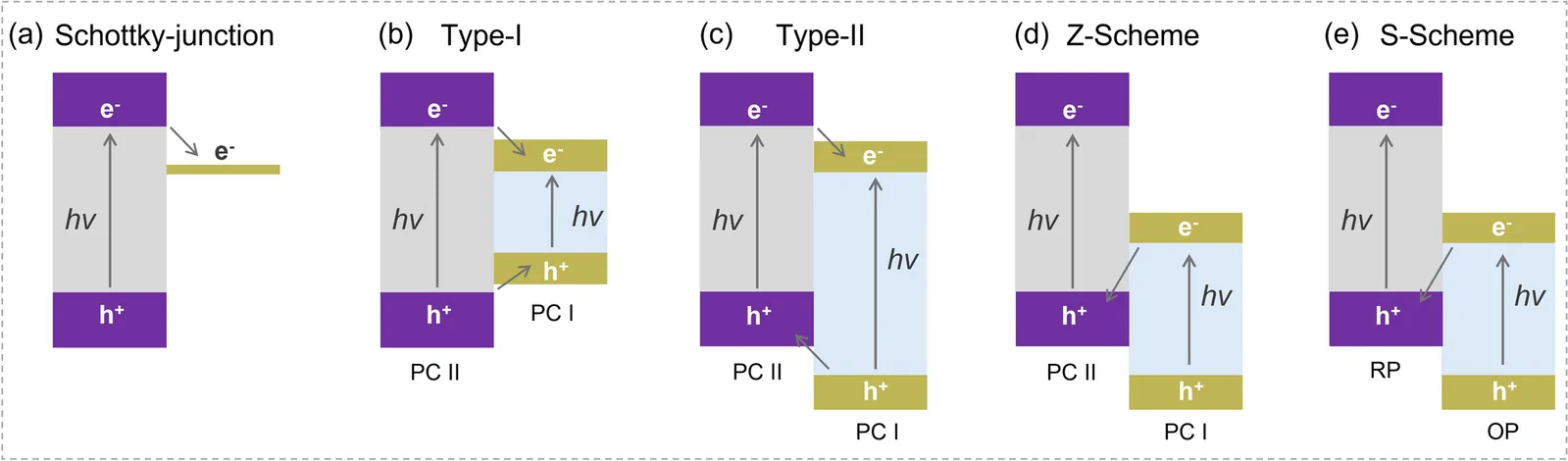
Schematic illustration of the band alignment and charge migration pathways in Schottky junction, Type-I, Type-II, Z-Scheme, and S-Scheme heterojunctions

Based on the above mechanism, the recombination of electron–hole pairs in the bulk or on the surface of photocatalysts would be competitive to the desired redox reactions, which is detrimental to photocatalytic performance. Construction of heterojunctions with intimate interfaces provides a promising approach to effectively separate the electron–hole pairs for redox reactions [30–32]. According to the alignment of energy levels and charge-transfer models, the reported MHPs-based heterojunctions can be divided into five types, including: (i) Schottky junction, (ii) Type-I, (iii) Type-II, (iv) Z-scheme, and (v) S-scheme (Fig. 4**)**. It will be discussed in detail in the following section.

### Categories of MHPs-Based Heterojunction Photocatalysts

In the past decades, considerable efforts have been made on the design and fabrication of heterojunctions to improve photocatalytic performance of MHPs. Overall, there are five typical categories of heterojunction photocatalysts, including: (i) Schottky junction, (ii) Type-I, (iii) Type-II, (iv) Z-scheme, and (v) S-scheme. The design principle will be discussed in detail in the following.

#### Schottky Junction

**Fig. 5:**
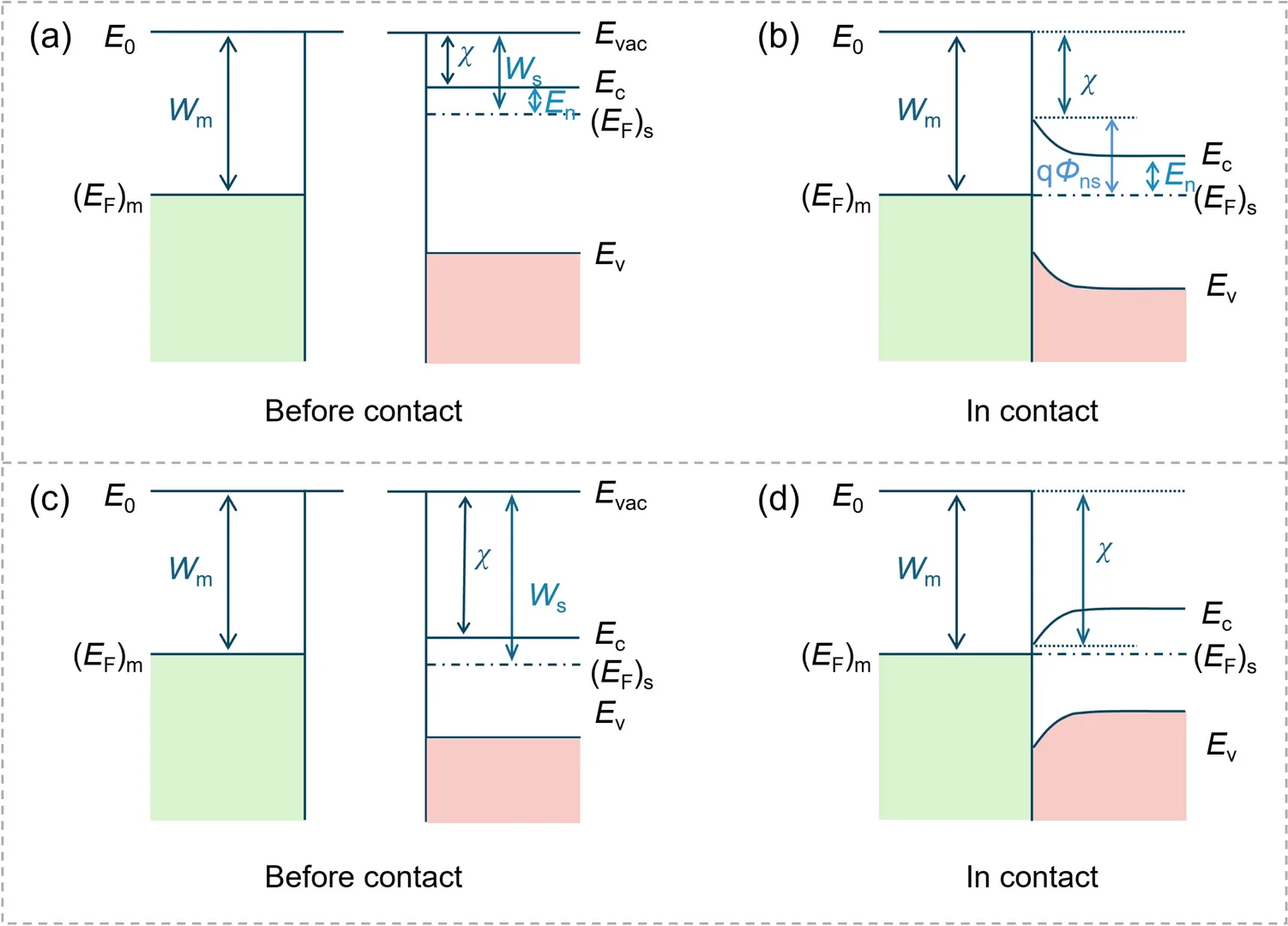
Schematic illustration of the energy band alignment of metal and n-type semiconductor contacts toward *W*_m_ > *W*_s_ **a** and **b**, and *W*_m_ < *W*_s_ **c** and **d**. *E*_vac_, vacuum energy; *E*_c_, energy of conduction band minimum; *E*_v_, energy of valence band maximum; *W*_m_, metal work function; *W*_s_, semiconductor work function; *χ*, electron affinity of the semiconductor

A Schottky junction is formed via the intimate contact of MHPs with a metal. Taking metal/n-type semiconductor as an example (Fig. 5) [32, 33], the differences in work functions (*W*) or Fermi level (*E*_*F*_) promote the free electrons transfer at the interface between the metal and semiconductor. When the Fermi level of semiconductor ((*E*_*F*_)_s_) is higher than that of metal ((*E*_*F*_)_m_), free electrons would flow from semiconductor to metal until the Fermi levels reach the same position, leading to the band bending and the formation of Schottky barrier. The back flow of electrons is effectively prevented by the Schottky barrier, and the high work function of metal provides a high driving force to transfer electrons to the adsorbed molecules. Under light irradiation, photogenerated electrons from the CB of MHPs would rapidly transfer to the metals to participate in the reduction reactions, while holes left in VB of MHPs would be consumed through oxidation reactions. On the contrary, when the Fermi level of semiconductor ((*E*_*F*_)_s_) is lower than that of metal ((*E*_*F*_)_m_), the electrons would transfer from metal to semiconductor, leading to the accumulation of electrons near semiconductor surface. Obviously, the lower (*E*_*F*_)_m_ than (E_*F*_)_s_ promotes the separation and transfer of photogenerated electron–hole under light irradiation, resulting the improved photocatalytic performance. For example, the loaded metal Pt with the highest work function (5.7 eV) and lowest Fermi level promoted H_2_ evolution activity of MAPbBr_3-x_I_x_ with ~ 3-time enhancement [34].

Few-layered metal chalcogenides (e.g., WS_2_) [35], transition metal carbide (e.g., Ti_3_C_2_) [36, 37], and carbon-based materials with good metallic conductivity, were also hybridized with MHPs to form a Schottky junction, like the semiconductor–metal heterojunction system. Benefiting from the following properties: (i) as a good electron acceptor and transfer channel, promoting electron–hole pairs separation [38], and (ii) large specific surface areas, providing sufficient active reaction sites, the formed Schottky junctions boost the photocatalytic performance of MHPs. For example, γ-CsPbI_3_ NCs hybridized with few-layered WS_2_ nanosheets exhibited a significant enhancement in photocatalytic methylene blue (MB) degradation, attributed to the increased amount of γ-CsPbI_3_ NCs and the superior carrier-transport property of few-layered WS_2_ nanosheets [35]. Moreover, DFT calculations on Ni_3_C/MAPbI_3_ revealed how metal work functioned and Fermi level alignment facilitated electron extraction and reduced recombination [39].

#### Type-I Heterojunction

**Fig. 6:**
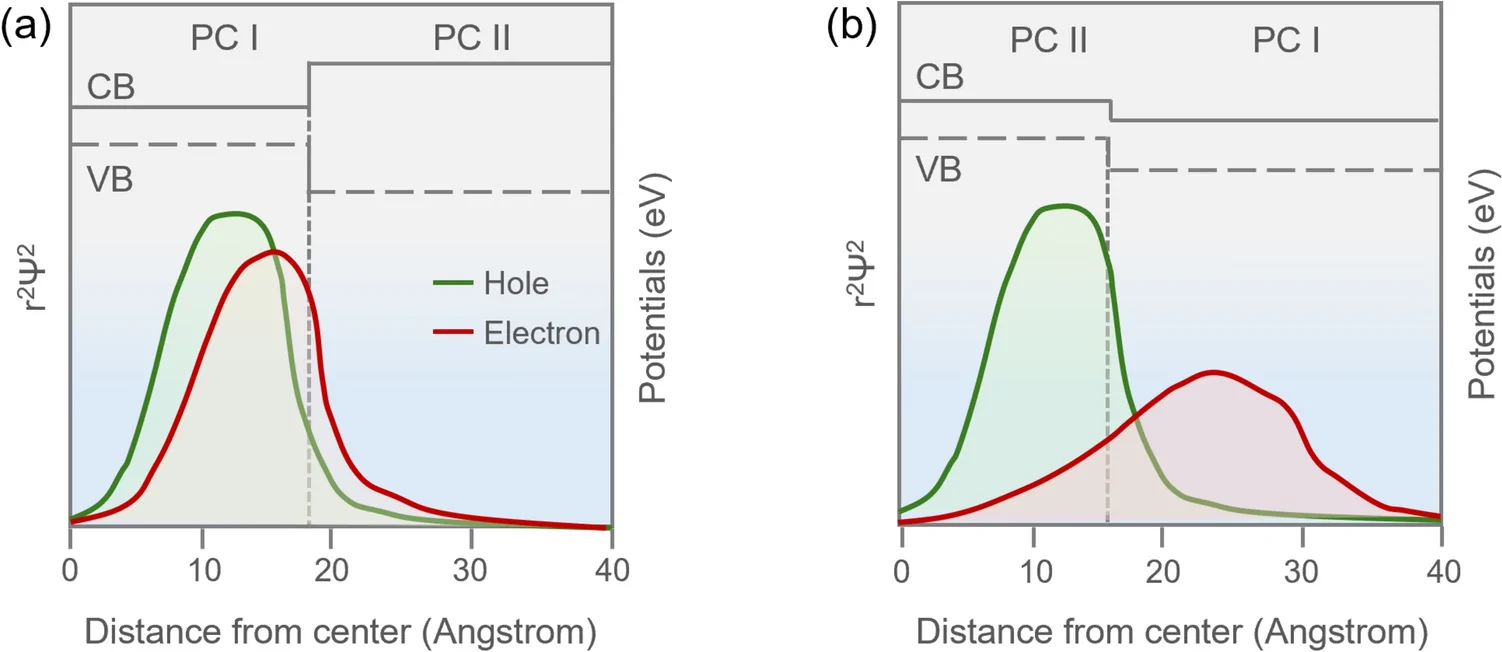
Schematic of radial distribution function of lowest energy conduction band (CB) electrons (red traces) and valence band (VB) holes (green lines) in **a** type-I and **b** type-II heterojunctions

The type-I heterojunction is formed by two coupled semiconductors (i.e., photocatalysts I and II) with straddling band structures. The CB energy potential of photocatalyst I (PC I) is lower than that of photocatalyst II (PC II), while VB potential of PC I is higher than that of PC II (Fig. 4b). Under light irradiation, the photogenerated electrons and holes in PC II would transfer to the CB and VB of PC I, respectively, resulting in the accumulation of both electrons and holes in PC I (Fig. 6a). Obviously, efficient spatial separation of photogenerated electron–hole pairs cannot be realized and the redox potentials of the heterojunction photocatalysts will be decreased, since both reduction and oxidation reactions occur on PC I with narrower bandgap [40]. However, the surface of PC I can be passivated with PC II, and more active sites would be exposed. For example, the constructed type-I heterojunction CsPbBr_3_/Cs_4_PbBr_6_ enhanced CO_2_ photoreduction activity toward CO [41]. In addition, MHPs nanocrystals immobilized on two-dimensional (2D) black phosphorus (BP) nanosheets promoted CO_2_ conversion rate because the introduced BP could offer more active sites for CO_2_ activation [42].

#### Type-II Heterojunction

One of the most popular heterojunctions is type-II, in which the CB and VB energy positions of PC II are higher than those of PC I with the staggered band structure (Fig. 4c). Upon light irradiation, photogenerated electrons on the CB of PC II with a higher reduction potential could transfer to PC I and holes in PC I with stronger oxidation ability would simultaneously transfer to PC II. Correspondingly, electron and hole wave functions can be preferentially localized in PC I and PC II, respectively (Fig. 6b) [43]. The decreased electron–hole overlap prolongs the lifetime of the exciton while decreases the exciton spin relaxation rate and non-radiative recombination, leading to effective spatial separation of photogenerated electron–hole pairs. Besides, the staggered band arrangement can extend the light absorption range through indirect transition between the two different materials’ energy levels [44]. The above properties enable MHPs-based type-II structure widely used in photocatalytic field. For example, the photocatalytic C(*sp*^3^)-H transformation activity of Cs_3_Bi_2_Br_9_/CdS heterojunction was much greater than those of bare Cs_3_Bi_2_Br_9_ and CdS owing to the staggered band structure [45], revealed by DFT calculations. Similarly, the in situ prepared Cs_2_SnI_6_/SnS_2_ showed a superior CO_2_ photoreduction to CO because of the ultrafast carrier separation and prolonged lifetime of electrons [21].

#### Z-Scheme Heterojunction

**Fig. 7:**
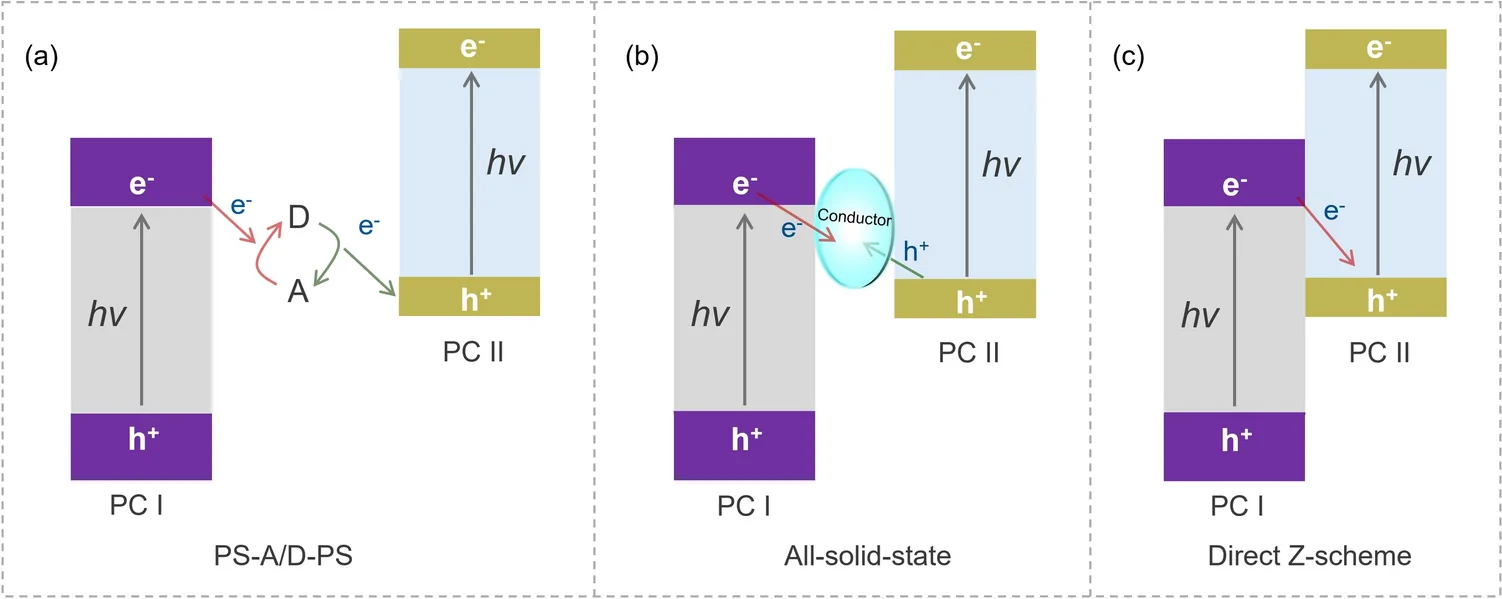
Illustration of three kinds of Z-scheme heterojunction structures, **a** PS-A/D-PS, **b** all-solid-state, and **c** direct Z-scheme

Although the charge-carrier separation can be promoted by type-II heterojunctions, the redox ability of photogenerated electron–hole is greatly weakened. In addition, from a dynamic perspective, the repulsion from the existing electrons in PC I will hinder the continuous transfer of electrons from PC II, impeding the realization of spatial charge separation. To overcome these drawbacks, the Z-scheme heterojunction has been introduced (Fig. 4d). According to composition, Z-scheme could be classified into three different structures [43, 46–48]: (i) PS-A/D-PS (Fig. 7a) with ionic redox pairs as ionic electron mediator (electron acceptor/donor pair known as A/D pair); (ii) all-solid-state Z-scheme with a solid conductor (e.g., metal, carbon nanotubes, graphene) as the mediator (Fig. 7b), and (iii) direct Z-scheme without mediator (Fig. 7c). MHPs-based Z-scheme junctions are mainly the last two structures. Under light irradiation, photogenerated electrons in CB of PC I would recombine with holes in the VB of PC II through the mediator with a low contact resistance interface. The solid conductor greatly shortens the distance of electron flow from PC I to PC II and renders a more promising electron relaying ability. For direct Z-scheme, the solid–solid contact interface allows Z-schematic vectorial electron transfer between semiconductors. The electrons in PC I would shuttle to PC II and combine with holes in PC II driven by strong electrostatic attraction between opposite charge clouds. Meanwhile, photoexcited holes in PC I and electrons in PC II would stay still because of the electrostatic repulsion. As a result, the photogenerated electrons and holes accumulate at the high potential to realize spatial separation and strong redox ability simultaneously. Taking advantage of this merit, Xu et al. constructed Cs_2_AgBiBr_6_@g-C_3_N_4_ Z-scheme system in toluene through an in situ assembly method to enhance CH_4_ generation and selectivity in photocatalytic CO_2_ reduction [49].

#### S-Scheme Heterojunction

**Fig. 8:**
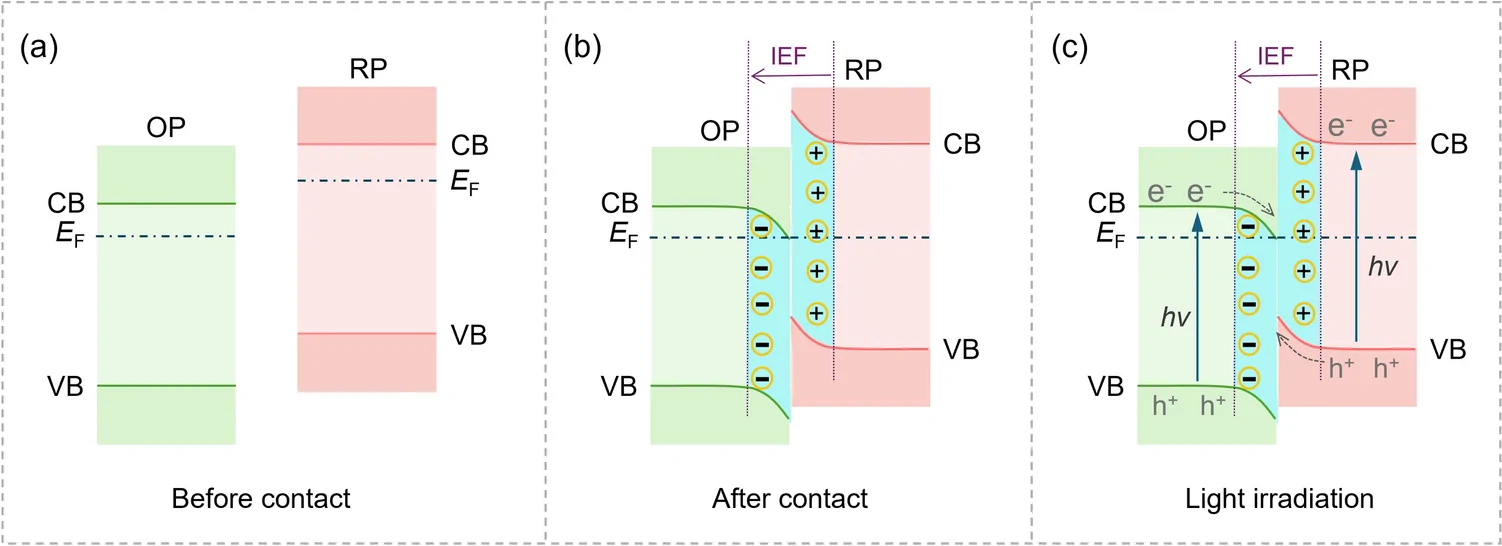
Schematic illustration of S-scheme heterojunction: internal electric field (IEF)-induced charge transfer, separation, and the formation of S-scheme heterojunction under light irradiation

Recently, S-scheme (or Step-scheme) heterojunction has been proposed, which consisted of RP (reduction photocatalyst with high CB) and OP (oxidation photocatalyst with low VB) with staggered band structure (Fig. 8a), like type-II heterojunction but with a different charge transfer route [50–52]. When they are in close contact (Fig. 8b), electrons in RP would spontaneously diffuse to OP, leading to the formation of electron depletion layer and electron accumulation layer in the interface of RP and OP, respectively. Consequently, an internal electric field directing from RP to OP would be created. Upon light irradiation (Fig. 8c), the band bending will drive the photogenerated electrons in the CB of OP and holes in the VB of RP to recombine at the interface. Photogenerated holes are reserved in the VB of OP and electrons are reserved in CB of RP, which makes the heterojunction have the highest oxidation–reduction capacity. For example, a fabricated 2D/2D BiVO_4_/CsPbBr_3_ S-scheme heterojunction through an in situ face-to-face grown strategy featured desirable accelerated dynamic carrier mobility, achieving high CO_2_-to-CO conversion with a turnover number (TON) near 230 without any co-catalyst or sacrificial agent [53]. DFT calculations revealed that such enhancement was attributed to the built-in electric fields and band bending in interfacial.

### Strategies for Building MHPs-based Heterojunction Photocatalysts

The rational design and synthesis of MHPs-based heterojunctions is of importance for photocatalytic performance. Various methods have been developed to fabricate MHPs-based heterojunction structures. Common preparation methods of MHPs-based heterojunction photocatalysts are summarized into the following four types.

#### Physical Mixing Method

Physical mixing is the simplest method to construct MHPs-based heterojunctions. In a typical procedure, the as-prepared MHPs were mixed with an appropriate amount of heteromaterials in solution and then ultrasonicated or stirred to allow the full hybridization through weak van der Waals interaction. Employing this simple and facile method, Bi_2_WO_6_ [54], P3HT conducting polymer [55], BiOBr [56], AgBr [57], Ti_3_C_2_ [37], and other materials [58, 59] were decorated onto MHPs to construct MHPs-based heterojunctions for solar-light-driven redox reactions.

#### Electrostatic Self-Assembly Method

Because of the weak van der Waals interaction, charge transfer only takes place when a free collision occurs, making the charge transfer random and inefficient. In this regard, an electrostatic self-assembly approach was developed to form intimate contact [16, 39]. For this process, the electrostatic interaction between oppositely charged semiconductors provided the driving force. For instance, 2D/2D CsPbBr_3_/BiOCl heterojunction was fabricated through electrostatic self-assembly of positively charged CsPbBr_3_ with Zeta-potentials of 17.8 mV and negatively charged BiOCl with Zeta-potential of -10.4 mV [60]. Because of the opposite charge on the surface, the assembly of CsPbBr_3_ and BiOCl would be spontaneous via electrostatic interaction. Similarly, positively charged CsPbBr_3_ was immobilized onto the negatively charged covalent triazine frameworks to fabricate type-II heterojunction for photocatalytic CO_2_ reduction to CO [61].

#### In situ* Growth Method*

In situ growth means that the as-prepared material is used as the substrate for the subsequent growth of another material or simultaneous growth of two materials in their mixed precursor solutions. According to the growth sequences, we classified this method into the following three types in detail.

*Addition of heteromaterials into MHPs precursor solution:* The heterojunction can be built by the growth of MHPs on the as-prepared heteromaterials. For instance, Li et al. applied in situ hot-injection method to synthesize CsPbBr_3_-CdZnS NRs heterojunction for CO_2_ photoreduction [62]. The strong affinity between S and Pb atoms enabled the pre-adsorption of Pb^2+^ on CdZnS. Cs precursor was then injected into the above mixture to grow CsPbBr_3_ onto CdZnS to form type-II CsPbBr_3_-CdZnS heterojunction. FAPbBr_3_/Bi_2_WO_6_ composite was also prepared by the antisolvent precipitation method, in which the precursor solution of FAPbBr_3_ was added dropwise to the suspension of as-prepared Bi_2_WO_6_ [63]. A mechanochemical synthesis with ball milling was also reported to grow MHPs onto heteromaterials. For example, DMASnBr_3_@g-C_3_N_4_ composite was constructed by adding g-C_3_N_4_ into DMABr and SnBr_2_, which were charged under inert atmosphere in a jar and milled for several hours [64].

*Addition of MHPs into heteromaterials precursor*: The heterojunctions are built by the growth of other semiconductors onto MHPs. To prepare CsPbBr_3_@ZIFs, the as-prepared CsPbBr_3_ QDs were first dispersed into ethyl acetate solution, and zinc acetylacetonate along with 2-methylimidazole was then added with constant stirring for ZIF growth [65]. In this process, the assistance of light, microwave, or thermal was usually introduced. For instance, Pt-SA/CsPbBr_3_ hybrid NCs were obtained through the introduction of monodisperse CsPbBr_3_ NCs into Pt precursor (platinum(II) bis-(acetylacetonate)) under light irradiation [66]. To synthesize CsPbBr_3_@GDY hybrid [67], the as-prepared CsPbBr_3_ nanocrystals as a growth substrate were mixed with hexamethylbenzene monomer in a microwave reactor. CsPbCl_3_/W_18_O_49_ composites were prepared by adding CsPbCl_3_ to WCl_6_ propanol solution through hydrothermal methods [68].

*Mixing of precursors of MHPs and heteromaterials*: The construction of heterojunctions which share the same metal by the two materials involves the mixing of precursors of MHPs and heteromaterials, such as the synthesis of MA_3_Bi_2_I_9_/DMA_3_BiI_6_ heterojunction [69]. In detail, the precursors including MAI, Bi(NO_3_)_3_, and HI were mixed and then transferred into a Teflon-lined stainless-steel autoclave for a solvothermal route. Employing this method, 0D CsPbBr_3_/2D CsPb_2_Br_5_ was also developed by hot injection using PbBr_2_ and Cs_2_CO_3_ as precursors [70]. Obviously, this approach ensures tight interfacial contact because of the formation of strong chemical bond between two materials, leading to effectively promoted charge-carrier dynamics.

#### Hybrid Strategies

To develop MHPs-based heterojunction photocatalysts with abundant properties, the above-mentioned strategies are usually combined collaboratively. For example, in situ growth was combined with physical mixing strategy to develop a ternary heterojunction material bismuth perovskite-TiO_2_-Ru(II) polypyridyl (CBB/TiO_2_/RuPS) [71]. In detail, CBB was first synthesized via ligand-assisted solvent reprecipitation method. TiO_2_ was then deposited onto CBB to obtain CBB/TiO_2_ by in situ hydrolysis. RuPS was finally anchored onto TiO_2_ shell by physical mixing of as-prepared RuPS and CBB/TiO_2_ in methanol under inert atmosphere. CsPbBr_3_/USGO/α-Fe_2_O_3_ hybrids were also constructed using in situ growth combining with electrostatic self-assembly approach [72]. However, it is still challenging for hybrid strategies, since multiple materials must be integrated in one at nanometer scale.

## MHPs-Based Heterojunctions for Solar-Light-Driven Redox Reactions

In recent years, solar-light-driven redox reactions by MHPs-based heterojunctions have achieved rapid development. In this part, we will discuss the progress of MHPs heterojunctions in terms of photocatalytic H_2_ evolution, CO_2_ reduction reaction, organic pollutant degradation, and organic synthesis reaction.

### MHPs-Based Heterojunctions for Photocatalytic H_2_ Evolution

Hydrogen (H_2_) with high-energy density and environmental benignity is considered as an ideal energy carrier to replace traditional fossil fuels [73, 74]. Solar-light-driven water splitting is a promising approach to produce renewable H_2_, in which solar energy is converted to chemical energy with two main redox processes, water oxidation for oxygen evolution and proton reduction for hydrogen evolution [75, 76]. However, it is still challenging to use MHPs as photocatalysts to generate H_2_ from water splitting, because of their ionic nature and poor stability in water. To overcome this issue, hydrohalic acid (HX) splitting as an alternative strategy was adopted for photocatalytic H_2_ evolution. In this strategy, MHPs were stabilized through a dynamic precipitation–solubility equilibrium in saturated HX solution. Compared to the four-electron water splitting, only two electrons were involved for HX splitting and the potential of X^−^ oxidation (e.g., E_I3-/I-_ = 0.53 V *vs.* NHE, pH = 0) was much lower than that of water oxidation (E_O2/H2O_ = 1.23 V *vs.* NHE, pH = 0). Table 1 summarizes the typical examples of H_2_ generation based on MHPs-based heterojunction photocatalysts.

**Table 1:** A summary of the photocatalytic activity of the representative MHPs-based heterojunctions in H_2_ evolution

Photocatalysts	Hetero-junction	Reaction solution	Electron donor	Light source	H_2_ generation rate	AQY (%)	Refs
PtSA/Cs_2_SnI_6_	Schottky junction	HI/H_3_PO_2_	HI	300 W Xe lamp, > 420 nm	430 μmol h^−1^ g^−1^	/	[77]
MAPbBr_3-x_I_x_/Pt	Schottky junction	HI/HBr/H_3_PO_2_	HI	300 W Xe lamp, > 420 nm	2604.8 μmol h^−1^ g^−1^	8.10 (450 nm)	[34]
MAPbI_3_/CoP	Schottky junction	HI/H_3_PO_2_	HI	300 W Xe lamp, > 420 nm	785.9 µmol h^−1^ g^−1^	/	[78]
Pt-DA_3_BiI_6_	Schottky junction	DAI/HI/H_3_PO_2_	HI	white LED lamp	57 μmol h^−1^ g^−1^	0.83 (535 nm)	[79]
Cs_2_AgBiBr_6_/N–C-140	Schottky junction	HBr/H_3_PO_2_	HBr	300 W Xe lamp, > 420 nm	380 μmol g^−1^ h^−1^	0.59 (420 nm)	[80]
MAPbI_3_-Ti_3_C_2_T_x_	Schottky junction	HI/H_3_PO_2_	HI	white LED lamp	578.2 μmol h^−1^ g^−1^	/	[37]
MAPbI_3_/rGO	Schottky junction	HI/H_3_PO_2_	HI	300 W Xe lamp, > 420 nm	894.3 μmol h^−1^ g^−1^	1.5 (450 nm)	[81]
Ni_3_C/MAPbI_3_	Schottky junction	HI/H_3_PO_2_	HI	300 W Xe lamp, > 420 nm	2362 μmol g^−1^ h^−1^	16.6 (420 nm)	[39]
BP/MAPbI_3_	Type-I	HI/H_3_PO_2_	HI	300 W Xe lamp, > 420 nm	3742 μmol h^−1^ g^−1^	23.2 (420 nm)	[82]
Cs_3_Bi_2_Br_9_/g-C_3_N_4_	Type-II	Water/triethanolamine	Triethanolamine	1500 W Xe lamp, 300–800 nm	1050 μmol g^−1^ h^−1^	/	[83]
MA_3_Bi_2_I_9_/DMA_3_BiI_6_	Type-II	HI/H_3_PO_2_	HI	300 W Xe lamp, > 420 nm	198.2 μmol h^−1^ g^−1^	/	[69]
ML-MoS_2_/MAPbI_3_-MC	Type-II	HI/H_3_PO_2_	HI	300 W Xe lamp, > 420 nm	3.6 mmol g^−1^ h^−1^	11.6 (450 nm)	[84]
Pt/TiO_2_-MAPbI_3_	Type-II	HI/H_3_PO_2_	HI	300 W Xe lamp, > 420 nm	1986.7 μmol h^−1^ g^−1^	70 (420 nm)	[85]
DMASnBr_3_@g-C_3_N_4_	Type-II	Water/triethanolamine	Triethanolamine	1500 W Xe lamp, 300–800 nm	18.6 μmol g^−1^ h^−1^	/	[64]
*V*_Br_-Cs_2_AgBiBr_6_/WO_3_	S-scheme	HBr/H_3_PO_2_	HBr	300 W Xe lamp, > 420 nm	364.89 µmol g^−1^ h^−1^	/	[86]

#### ***Schottky Junctions for Photocatalytic H***_***2***_*** Evolution***

**Fig. 9:**
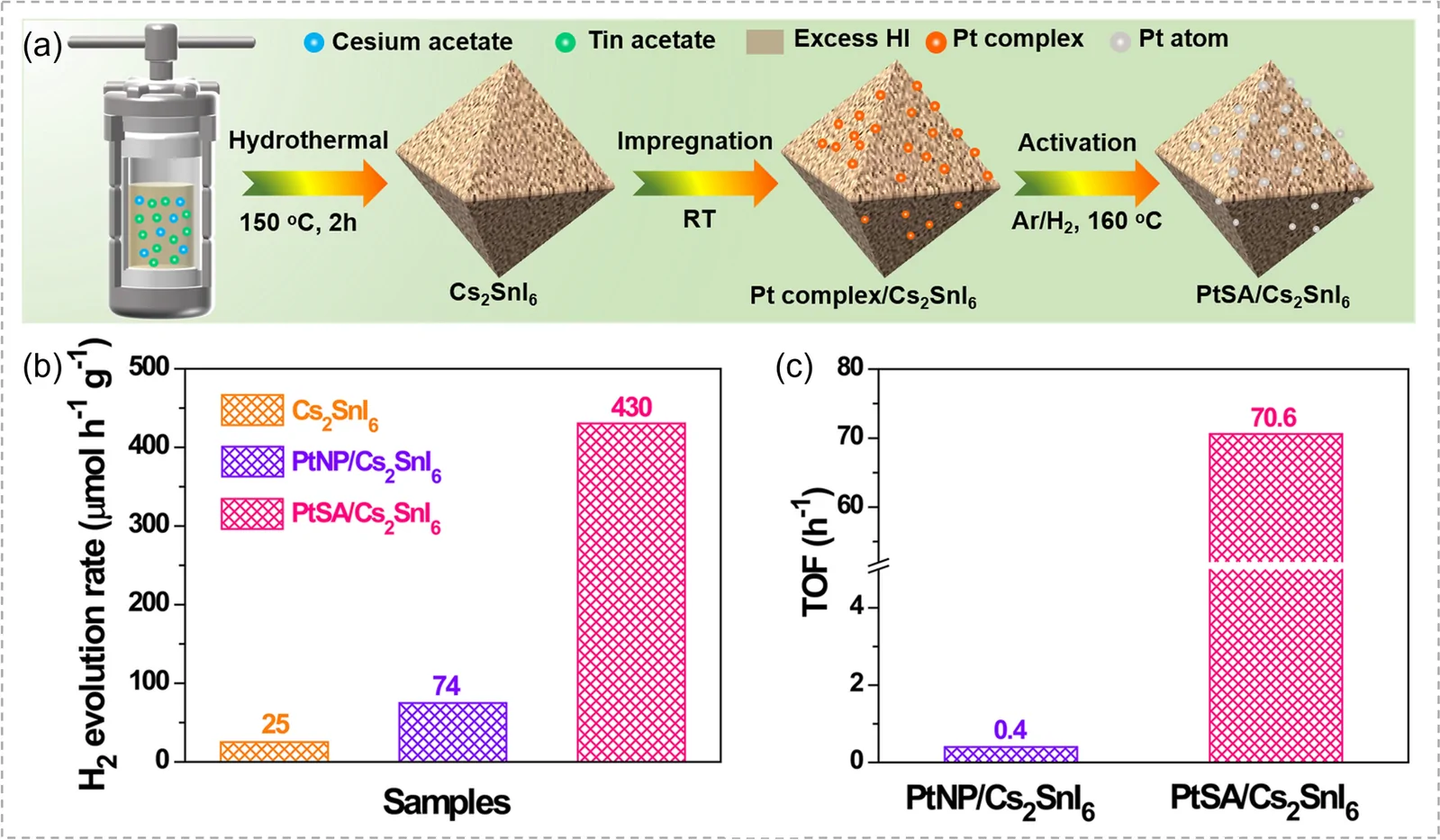
**a** Schematic illustration of PtSA/Cs_2_SnI_6_ preparation process. **b** Photocatalytic H_2_ generation rate over PtSA/Cs_2_SnI_6_, PtNP/Cs_2_SnI_6_, and Cs_2_SnI_6_ catalysts, and **c** the corresponding TOF values of PtSA/Cs_2_SnI_6_ and PtNP/Cs_2_SnI_6_ catalysts. Reproduced with permission [77]. Copyright 2020, Springer Nature

The first work on MHPs-based heterojunction for H_2_ evolution was reported in 2016 by Nam and coworkers [11], in which Pt nanocrystals were loaded on MAPbI_3_ via in situ photoreduction. The modification of Pt not only formed a Schottky junction, but also acted as a cocatalyst for proton reduction. The resulting MAPbI_3_/Pt showed a 1.8-fold enhancement for H_2_ evolution activity. Following this pioneering work, Pt nanoparticles and single atoms were widely decorated to construct Schottky junction with MHPs [34]. Because of serious environmental issues, the lead-free MHPs were recently developed to construct Schottky junction, such as (CH_3_NH_3_)_3_Bi_2_I_9_/Pt [87], and PtSA/Cs_2_SnI_6_ [77], to improve photocatalytic H_2_ evolution activity (Fig. 9).

**Fig. 10:**
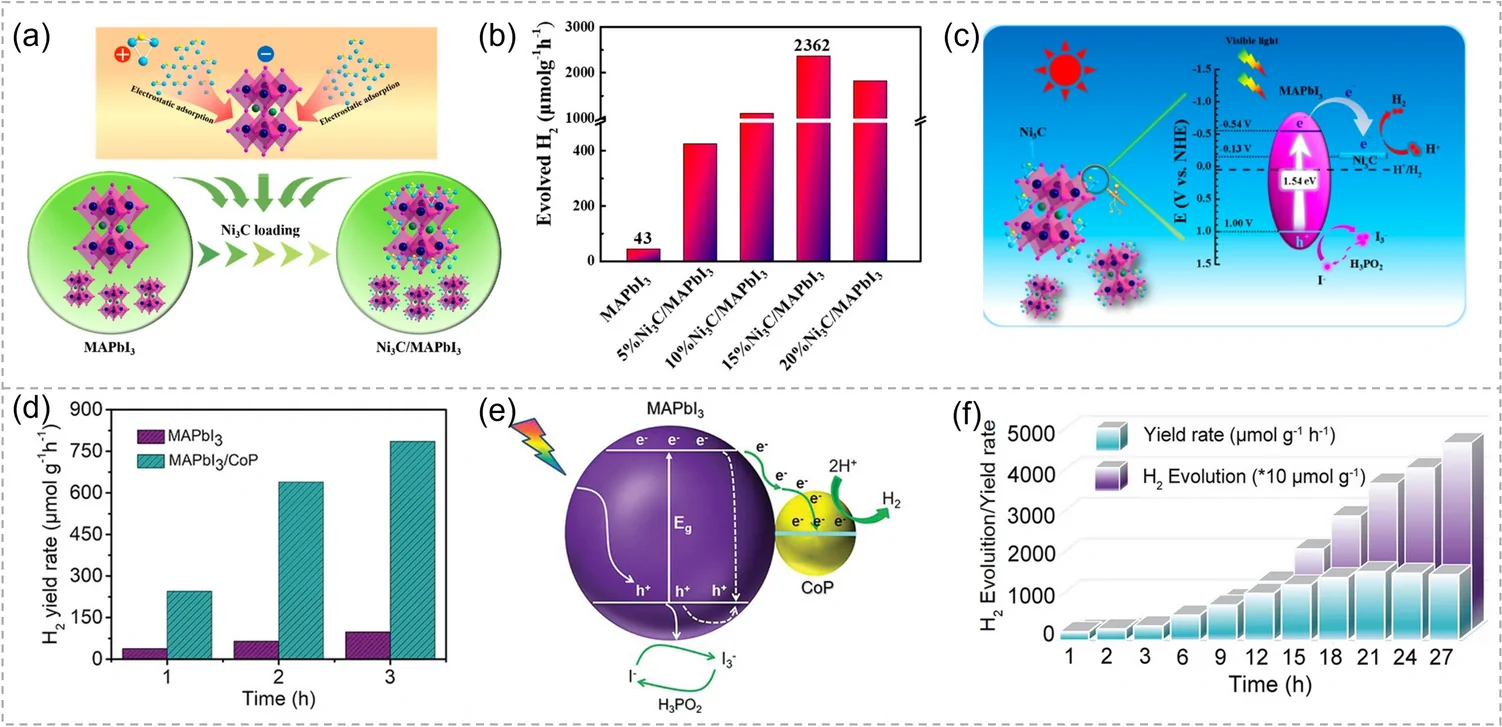
**a** Schematic illustration of synthesis process of Ni_3_C/MAPbI_3_ photocatalyst, **b** photocatalytic H_2_ evolution performance under light irradiation, and **c** band diagram of Ni_3_C/MAPbI_3_ for HI splitting reaction. Reproduced with permission [39]. Copyright 2019, American Chemical Society. **d** Photocatalytic H_2_ evolution rate of pristine MAPbI_3_ and MAPbI_3_/CoP hybrid, **e** schematic illustration of H_2_ generation process, and **f** stability test over MAPbI_3_/CoP hybrid in MAPbI_3_-saturated HI solution. Reproduced with permission [78]. Copyright 2020, Wiley–VCH

Besides, the materials with metallic character were also incorporated to construct Schottky junctions to improve photocatalytic H_2_ evolution. For example, Ni_3_C/MAPbI_3_ photocatalyst was synthesized by a facile surface charge-promoted self-assembly route (Fig. 10a) [39]. Owing to the improved charge-carrier transfer and separation as well as the massive reactive centers, the optimal Ni_3_C/MAPbI_3_ photocatalyst (2362 μmol g^−1^ h^−1^) showed almost 55-fold improvement of H_2_ generation rate in HI saturated aqueous solution (Fig. 10b, c) compared to pristine MAPbI_3_ (43 μmol g^−1^ h^−1^). Likewise, Min et al. obtained MAPbI_3_/Ti_3_C_2_T_x_ through in situ integration of MAPbI_3_ microcrystals with Ti_3_C_2_T_x_ NSs [37]. The optimal MAPbI_3_/Ti_3_C_2_T_x_ hybrid exhibited a 43-fold enhancement of H_2_ evolution rate relative to that of pure MAPbI_3_. More importantly, a stable H_2_ evolution activity over a 120-h reaction period was achieved, due to the interfacial passivation effect of Ti_3_C_2_T_x_. CoP with excellent conductivity was also reported to construct MAPbI_3_/CoP Schottky junctions to boost photocatalytic H_2_ evolution performance of MHPs (Fig. 10d-f) [78].

#### ***Type-I Heterojunctions for Photocatalytic H***_***2***_*** Evolution***

**Fig. 11:**
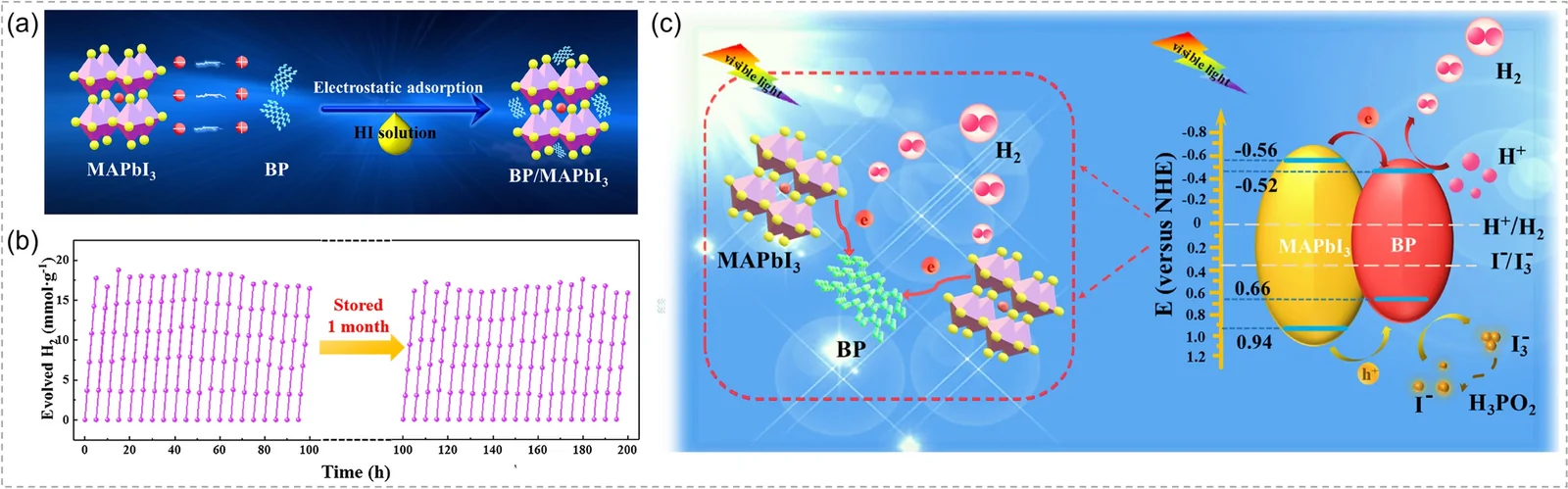
**a** Schematic diagram of BP/MAPbI_3_ preparation process, **b** cycling tests of H_2_ production, and **c** photogenerated charge transfer over BP/MAPbI_3_ under light illumination. Reproduced with permission [82]. Copyright 2019, Elsevier

Type-I heterojunctions were constructed by coupling MHPs and heteromaterials with suitable band alignment to facilitate H_2_ evolution. Chen et al. anchored 2D few-layer black phosphorus (BP) on MAPbI_3_ via electrostatic coupling to obtain a type-I heterojunction (Fig. 11) [82]. The mechanism insights indicated that BP not only extracted electrons from MAPbI_3_ through a type-I heterojunction, but also provided abundant active sites for proton reduction reaction. As a result, the BP/MAPbI_3_ composite yielded a superior H_2_ generation rate of 3742 μmol h^−1^ g^−1^. Because the electrostatic adsorbed BP on the surface of MAPbI_3_ resulted in interfacial passivation, the composite showed an excellent stability in HI solution. The lead-free MHPs were also adopted to form type-I heterojunction, because of their environmentally friendly behavior. For example, Zhao et al. synthesized NiCoP/Cs_2_AgBiBr_6_ (NCP/CABB) via a simple electrostatic adsorption method [88]. Benefiting from the broadened visible light absorption range and promoted photoelectrons transfer, NCP/CABB achieved an 88-time improvement of H_2_ generation.

#### ***Type-II Heterojunctions for Photocatalytic H***_***2***_*** Evolution***

**Fig. 12:**
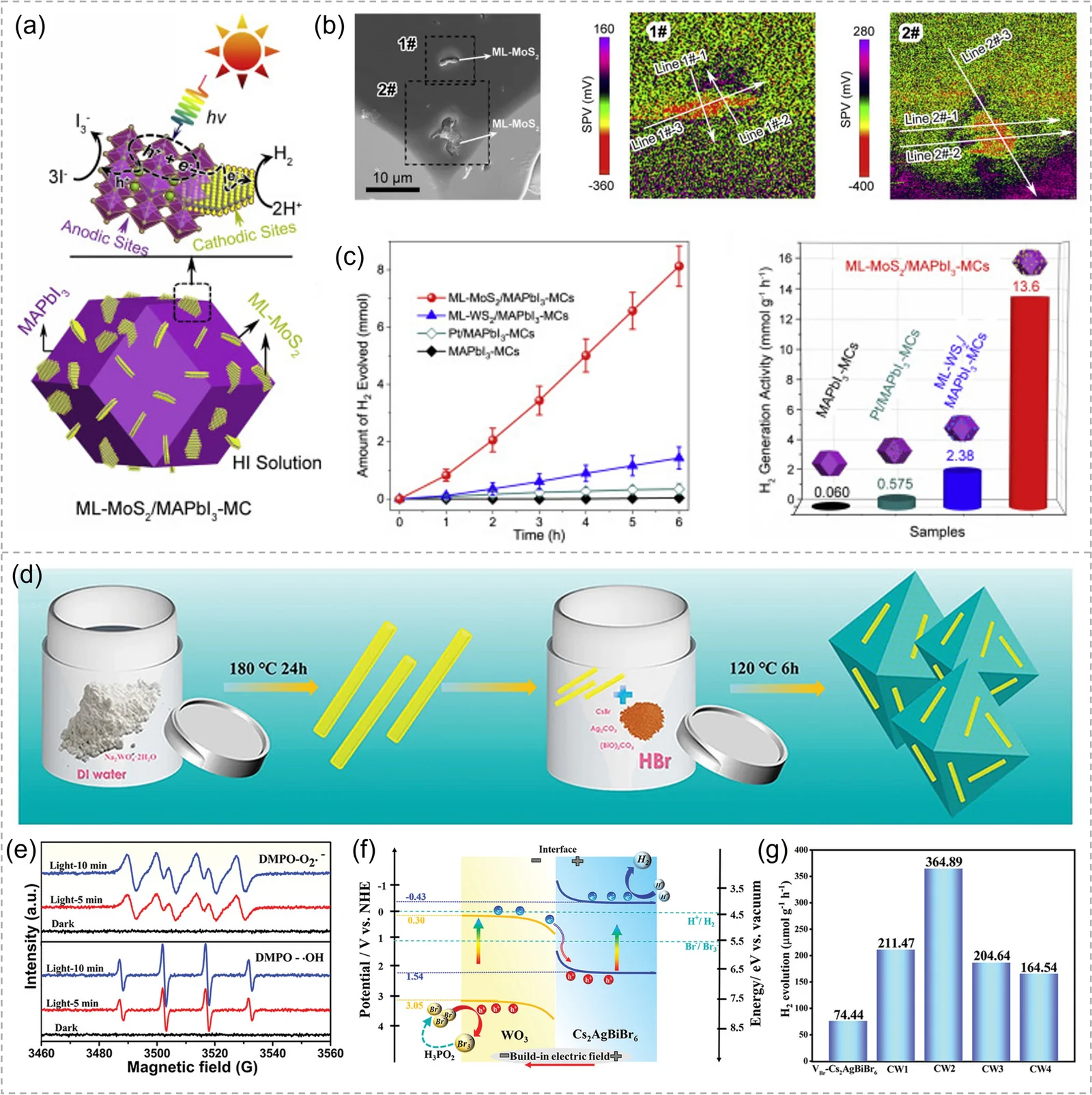
**a** Schematic illustration of structural configuration and redox processes, **b** SPV distribution of ML-MoS_2_/MAPbI_3_-MCs, and **c** photocatalytic HI splitting performance over ML-MoS_2_/MAPbI_3_-MCs, ML-WS_2_/MAPbI_3_-MCs, Pt/MAPbI_3_-MCs, and the pristine MAPbI_3_-MCs. Reproduced with permission [84]. Copyright 2020, Elsevier. **d** Schematic illustration of *V*_Br_-Cs_2_AgBiBr_6_/WO_3_ fabrication process, **e** EPR spectra of DMPO- ⋅O_2_^−^ and DMPO-⋅OH before and after light irradiation, and **f** schematic mechanism of the charge transfer and dynamic equilibrium processes over *V*_Br_-Cs_2_AgBiBr_6_/WO_3_ composite, and **g** H_2_ production rate of *V*_Br_-Cs_2_AgBiBr_6_/WO_3_, and CW_x_ in aqueous HBr/H_3_PO_2_ solutions. Reproduced with permission [86]. Copyright 2023, Wiley–VCH

Owing to the efficient charge-carrier spatial separation, type-II heterojunction has aroused great attention for enhanced photocatalytic H_2_ production. Zhao et al. anchored small-sized monolayer MoS_2_ nanosheets (ML-MoS_2_), onto large-sized MAPbI_3_ microcrystal (MAPbI_3_-MC) to construct type-II heterojunction MAPbI_3_-MC/ML-MoS_2_ [84]. The surface photovoltage differences in Kelvin probe force microscopy directly confirmed the effective spatial separation of the photogenerated electrons and holes, and the existence of strong built-in electric field aligned between MAPbI_3_-MC (oxidation site) and ML-MoS_2_ (reduction site) (Fig. 12a, b). A superior H_2_ production rate of 13.6 mmol g^−1^ h^−1^ was achieved under visible light (Fig. 12c). MoSe_2_ has also been reported to couple with MA_1-x_FA_x_PbI_3_ to construct type-II heterojunction for enhanced H_2_ evolution [89]. Similarly, a type-II heterogeneous photocatalyst Cs_3_Bi_2_Br_9_/g-C_3_N_4_ with the positive band-alignment led to an efficient charge separation [83]. Interestingly, a strategy by sharing a common metal to construct type-II heterojunction based on lead-free MHPs, MA_3_Bi_2_I_9_/DMA_3_BiI_6_ [69], was recently successfully developed with a solvent engineering technique. An enhanced photoinduced charge separation was achieved with a prolonged exciton lifetime of ~ 38 ns for the hybrid MA_3_Bi_2_I_9_/DMA_3_BiI_6_, leading to an improvement of the solar-light-driven H_2_ evolution efficiency.

#### ***Z-Scheme Heterojunctions for Photocatalytic H***_***2***_*** Evolution***

Z-scheme heterojunction has also been constructed to improve photocatalytic H_2_ evolution performance of MHPs. For instance, Zeng et al. anchored two-dimensional black phosphorus (BP) onto Cs_2_AgBiBr_6_ (CABB) through electrostatic coupling [90]. The resulted Z-scheme BP/CABB showed a higher H_2_ generation rate of 104.6 µmol g^−1^ h^−1^ than that of bare CABB, attributed to the key role of BP as an electronic accelerator.

#### ***S-Scheme Heterojunctions for Photocatalytic H***_***2***_*** Evolution***

Different from traditional type-II heterojunction, S-scheme heterojunction has been proved to maintain the strong redox potential. Zhang et al. constructed an S-scheme heterojunction of Cs_2_AgBiBr_6_ regular octahedron crystals with enriched Br-vacancies and WO_3_ nanorods (*V*_Br_-Cs_2_AgBiBr_6_/WO_3_) (Fig. 12d) [86]. Owing to the fast electron transfer from WO_3_ to *V*_Br_-Cs_2_AgBiBr_6_, an increased and decreased electron density was observed on the surface of *V*_Br_-Cs_2_AgBiBr_6_, and WO_3_, respectively, indicating the formation of S-scheme heterojunction between WO_3_ and *V*_Br_-Cs_2_AgBiBr_6_. EPR tests indicated the generation of superoxide radical (⋅O_2_^−^) in *V*_Br_-Cs_2_AgBiBr_6_/WO_3_, attributed to the reduced O_2_. The presence of ⋅OH signal demonstrated that *V*_Br_-Cs_2_AgBiBr_6_/WO_3_ could oxidize H_2_O/OH^−^ to ⋅OH (Fig. 12e). All these results proved the formation of S-scheme heterojunction instead of type-II heterojunction (Fig. 12f). The optimal *V*_Br_-Cs_2_AgBiBr_6_/WO_3_ exhibited almost 4.9-fold higher photocatalytic H_2_ evolution activity than that of bare *V*_Br_-Cs_2_AgBiBr_6_ (Fig. 12g).

### MHPs-Based Heterojunctions for Photocatalytic CO_2_ Reduction

Photocatalytic CO_2_ reduction under ambient temperature to produce solar fuels or chemicals stands out as a dual-functional reaction in mitigating climate and energy crisis [91, 92]. In this process, CO_2_ molecules are first adsorbed and activated and then converted into various intermediate species through sequential hydrogenation and dehydration procedure [93, 94]. Multiple photogenerated electrons are involved in this process, generating various valuable products, such as CO, CH_4_, HCOOH, CH_3_OH, C_2_H_5_OH. The detailed steps and corresponding redox potentials are shown in Eqs. 1–5 [95, 96], which are all satisfied by the CB potential of MHPs. Since the first work reported by Sun’s group in 2017 [97], continuous efforts have been devoted (Table 2). In general, solar light absorption and charge-carrier separation and transport are two main factors in photocatalytic CO_2_ reduction systems. The design of MHPs-based heterojunctions is one of the most popular strategies to improve efficiency over these two factors.

1 $$\begin{array}{*{20}c} {{\mathrm{CO}}_{2} + 2{\mathrm{e}}^{ - } + 2{\mathrm{H}}^{ + } = {\mathrm{CO}} + {\mathrm{H}}_{2} {\mathrm{O}}} & {{\mathrm{E}}_{1} = - 0.53{\mathrm{V}}\,vs\,{\mathrm{NHE}}\,(pH = 7)} \\ \end{array}$$

2 $$\begin{array}{*{20}c} {{\mathrm{CO}}_{2} + 2{\mathrm{e}}^{ - } + 2{\mathrm{H}}^{ + } = {\mathrm{HCOOH}}} & {{\mathrm{E}}_{2} = - 0.61{\mathrm{V}}\,vs\,{\mathrm{NHE}}\,(pH = 7)} \\ \end{array}$$

3 $$\begin{array}{*{20}c} {{\mathrm{CO}}_{2} + 4{\mathrm{e}}^{ - } + 4{\mathrm{H}}^{ + } = {\text{HCHO + H}}_{{2}} {\mathrm{O}}} & {{\mathrm{E}}_{3} = - 0.48{\mathrm{V}}\,vs\,{\mathrm{NHE}}\,(pH = 7)} \\ \end{array}$$

4 $$\begin{array}{*{20}c} {{\mathrm{CO}}_{2} + 6{\mathrm{e}}^{ - } + 6{\mathrm{H}}^{ + } = {\mathrm{CH}}_{{3}} {\text{OH + H}}_{{2}} {\mathrm{O}}} & {{\mathrm{E}}_{4} = - 0.38{\mathrm{V}}\,vs\,{\mathrm{NHE}}\,(pH = 7)} \\ \end{array}$$

5 $$\begin{array}{*{20}c} {{\mathrm{CO}}_{2} + 8{\mathrm{e}}^{ - } + 8{\mathrm{H}}^{ + } = {\mathrm{CH}}_{{4}} {\text{ + 2H}}_{{2}} {\mathrm{O}}} & {{\mathrm{E}}_{5} = - 0.24{\mathrm{V}}\,vs\,{\mathrm{NHE}}\,(pH = 7)} \\ \end{array}$$

**Table 2:** A summary of the photocatalytic activity of the MHPs-based heterojunctions toward CO_2_ reduction

Photocatalysts	Hetero-junction	Reaction solution	Electron donor	Light Source	Products	Activity	Refs.
CsCuCl_3_/Cu	Schottky junction	Ethyl acetate	Isopropyl alcohol	simulated sunlight	CH_4_	21.61 μmol g^−1^	[98]
CsPbBr_3_ QDs/ GO	Schottky junction	Ethyl acetate	Ethyl acetate	AM 1.5G	CH_4_	29.6 μmol g^−1^	[97]
					CO	58.7 μmol g^−1^	
CsPbBr_3_/Bi	Schottky junction	None	Water	300 W Xe lamp	CO	76.4 μmol g^−1^	[58]
CsPbBr_3_ NC/Pd NS	Schottky junction	None	Water	300 W Xe lamp, > 420 nm	CH_4_	3.94 μmol g^−1^	[99]
					CO	12.63 μmol g^−1^	
CsPbBr_3_/ MWCNT	Schottky junction	Acetonitrile	Water	300 W Xe lamp, > 420 nm	CH_4_	33.5 μmol g^−1^	[100]
					CO	98.3 μmol g^−1^	
CsPbBr_3_/Ti_3_C_2_T_x_	Schottky junction	Ethyl acetate	Ethyl acetate	300 W Xe lamp, > 420 nm	CH_4_	33.83 μmol g^−1^	[101]
					CO	133.05 μmol g^−1^	
FAPbBr_3_/Ti_3_C_2_	Schottky junction	Water	Water	Simulated sunlight	CH_4_	17.67 μmol g^−1^ h^−1^	[102]
					CO	283.41 μmol g^−1^ h^−1^	
CsPbBr_3_/BP	Type-I	Ethyl acetate	Water	Simulated sunlight	CH_4_	32 μmol g^−1^	[42]
					CO	134 μmol g^−1^	
CsPbBr_3_@GDY-Co	Type-I	Acetonitrile	Water	300 W Xe lamp, > 400 nm	CO	27.7 μmol g^−1^ h^−1^	[67]
CsPbBr_3_/MoS_2_	Type-II	Ethyl acetate	Water	300 W Xe lamp, > 420 nm	CH_4_	54.7 μmol g^−1^	[103]
					CO	74.9 μmol g^−1^	
Cs_2_SnI_6_/SnS_2_	Type-II	H_2_O/CH_3_OH	CH_3_OH	300 W Xe lamp, > 400 nm	CH_4_	6.09 μmol g^−1^	[21]
CsSnCl_3_/ZnSe	Type-II	Toluene/ isopropanol	Isopropanol	300 W Xe lamp, > 400 nm	CO	55 μmol g^−1^ h^−1^	[16]
CsPbBr_3_-CdZnS	Type-II	None	Water	300 W Xe lamp	CO	55.8 µmol g^−1^ h^−1^	[62]
CsPbBr_3_-P3HT	Type-II	Acetonitrile	Water	300 W Xe lamp, > 420 nm	CO	145.45 μmol g^−1^ h^−1^	[55]
					CH_4_	23.05 μmol g^−1^ h^−1^	
Cs_2_AgBiBr_6_/ Ce-UiO-66-H	Type-II	None	Water	300 W Xe lamp	CO	309.01 μmol g^−1^ h^−1^	[104]
g-C_3_N_4_@ Cs_2_AgBiBr_6_	Type-II	Ethyl acetate/ water	Triethylamine	300 W Xe lamp, > 420 nm	CO	35.52 μmol g^−1^	[105]
					CH_4_	3.91 μmol g^−1^	
g-C_3_N_4_-CsPbBr_3_	Type-II	Acetonitrile	Water	300 W Xe lamp, > 420 nm	CO	149 μmol g^−1^	[106]
α-Fe_2_O_3_/Amine RGO/CsPbBr_3_	Z-scheme	None	Water	300 W Xe lamp, > 420 nm	CH_4_	9.45 μmol g^−1^ h^−1^	[107]
LF-FAPbBr_3_/α-Fe_2_O_3_	Z-scheme	None	Water	300 W Xe lamp, > 400 nm	CO	45.5 μmol g^−1^ h^−1^	[108]
					CH_4_	10.5 μmol g^−1^ h^−1^	
SnS_2_/CsPbBr_3_	Z-scheme	None	Water	300 W Xe lamp, 300–800 nm	CO	1.98 μmol g^−1^ h^−1^	[109]
CsPbBr_3_/ CsPb_2_Br_5_	Z-scheme	None	Water	300 W Xe lamp	CO	197.11 μmol g^−1^ h^−1^	[70]
CsPbBr_3_/NiFe-LDH	Z-scheme	Ethyl acetate/ isopropanol	Isopropanol	300 W Xe lamp, > 420 nm	CO	10.5 μmol g^−1^ h^−1^	[110]
					CH_4_	2.64 μmol g^−1^ h^−1^	
PCN-222/ CsPbBr_3_	Z-scheme	Acetonitrile	Triethylamine	300 W Xe lamp, > 420 nm	HCOOH	189.9 μmol g^−1^ h^−1^	[111]
Cs_3_Bi_2_I_9_/Bi_2_WO_6_	Z-scheme	None	Water	300 W Xe lamp, > 400 nm	CO	66 μmol g^−1^	[112]
Ni-doped CsPbBr_3_/Bi_3_O_4_Br	Z-scheme	None	Water	300 W Xe lamp	CO	387.57 μmol g^−1^ h^−1^	[113]
BiVO_4_/CsPbBr_3_	S-scheme	None	Water	300 W Xe lamp	CO	68 μmol g^−1^	[53]
CsPbBr_3_/BiOCl	S-scheme	Ethyl acetate	Water	300 W Xe lamp	CO	34.72 μmol g^−1^ h^−1^	[60]
					CH_4_	3.47 μmol g^−1^ h^−1^	
Cs_3_Bi_2_Br_9_/Bi-MOF	S-scheme	None	Water	300 W Xe lamp	CO	572.24 μmol g^−1^ h^−1^	[114]
Cs_3_Bi_2_Br_9_@M-Ti framework	S-scheme	Isopropanol	Isopropanol	300 W Xe lamp, 200–1100 nm	CH_4_	32.9 μmol g^−1^ h^−1^	[115]
SnO_2_/Cs_3_Bi_2_Br_9_	S-scheme	Acetonitrile	Water	300 W Xe lamp	CH_4_	21.4 μmol g^−1^	[116]

#### ***Schottky Junctions for Photocatalytic CO***_***2***_*** Reduction***

**Fig. 13:**
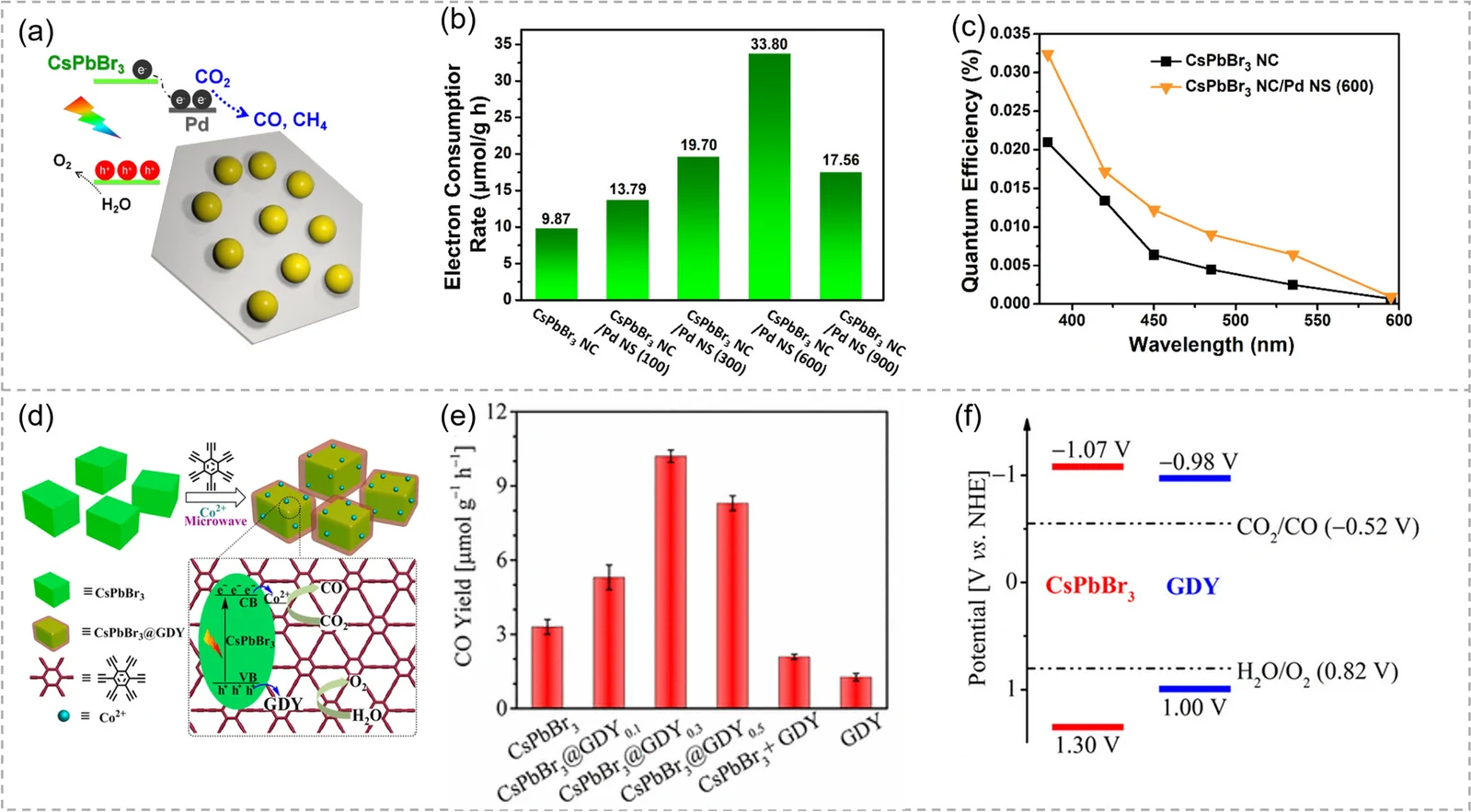
**a** Mechanism of CsPbBr_3_/Pd photocatalytic CO2 reduction, **b** the rate of electron consumption, and **c** the quantum efficiency of the hybrid under different wavelength. Reproduced with permission [99]. Copyright 2018, American Chemical Society. **d** Illustration of the cobalt-doped CsPbBr_3_@GDY preparation process, **e** CO production yield of CO_2_ photoreduction with CsPbBr_3_, CsPbBr_3_@GDY_0.1–0.5_, a mixture of CsPbBr_3_ and GDY, and GDY as photocatalysts, and **f** energy band alignment of CsPbBr_3_ and GDY. Reproduced with permission [67]. Copyright 2020, American Chemical Society

Metals are usually hybridized with MHPs to construct Schottky junction for CO_2_ reduction. For example, a novel zero-dimensional CsPbBr_3_ nanocrystal/two-dimensional palladium nanosheet (CsPbBr_3_ NC/Pd NS) composite was reported to realize CO_2_ reduction in H_2_O vapor (Fig. 13a-c) [99]. The Schottky contact at the interface extracted the photoinduced electrons from CsPbBr_3_ to Pd effectively, facilitating electrons injection into the subsequent chemical reactions. Su et al. encapsulated CsPbBr_3_ and Au particles by Al-based mesoporous metal–organic framework (MOF) to prepare a ternary CsPbBr_3_/Au/PCN-333(Al) hybrid composite [117]. The synergetic effect enabled by the Schottky contact between CsPbBr_3_ and Au in MOF matrix improved the photocatalytic CO_2_ reduction performance largely with the *R*_*electron*_ exhibiting 11.5-fold enhancement compared to single CsPbBr_3_ NCs. Moreover, the integrated PCN-333(Al) as a protective layer was used to encapsulate MHPs, leading to a good stability of the photocatalytic activity after 5 cycles.

2D MXene nanosheets (e.g., Ti_3_C_2_T_x_) have recently been widely used to construct Schottky heterojunctions with MHPs for CO_2_ reduction. Xing et al. presented a self-assembled heterojunction of lead-free double perovskite onto the surface of MXene (Cs_2_AgBiBr_6_/Ti_3_C_2_T_x_) via electrostatic attraction [36]. The presence of MXene not only accelerated the formation of free charge carriers, but also enabled the ultrafast photoelectrons transfer from Cs_2_AgBiBr_6_ to MXene within 1.1 ps and prolongs the charge-carrier lifetime. Following this work, various Schottky junction heterojunctions, such as CsPbBr_3_/Ti_3_C_2_T_x_ [118], FAPbBr_3_ QDs/Ti_3_C_2_ [102], CsPbBr_3_ QDs/Ti_3_C_2_ [101], for enhanced photocatalytic CO_2_ reduction were developed.

In addition, a composite of CsPbBr_3_ quantum dot/graphene oxide (CsPbBr_3_ QDs/GO) was obtained through in situ growth with the addition of GO into CsPbBr_3_ precursor [97]. Because of the improved electron extraction and transfer, CO_2_ reduction reached a rate of 23.7 μmol g^−1^ h^−1^ with an increased 25.5% electron consumption. Other carbon materials such as rGO [119], multiwalled carbon [100], C_60_ [120], nanoporous carbon power [121], etc., were also incorporated with MHPs to improve CO_2_ conversion performances.

#### ***Type-I Heterojunctions for Photocatalytic CO***_***2***_*** Reduction***

Zhu and coworkers reported the first MHPs-based type-I heterojunction CsPbBr_3_/BP for photocatalytic CO_2_ reduction [42]. Pb-P interaction in CsPbBr_3_/BP composite, revealed by the high-resolution XPS spectra, promoted the efficient transport of photogenerated electrons between excited CsPbBr_3_ and BP. 4.4- and 2.4-fold enhancements were achieved for CO_2_ photoreduction to CO and CH_4_, respectively, compared to single CsPbBr_3_. As a new member of the 2D material family, graphdiyne (GDY) was coated onto the surface of CsPbBr_3_ nanocrystals (Fig. 13d) [67]. Under the protection of GDY, the stability of MHPs-based composites in photocatalytic system was improved. The *sp*-hybridized carbon atoms and triangular cavities in the GDY accelerated the metal atom doping to act as the active sites for photocatalytic reaction (Fig. 13e). The favorable energy alignment and close contact triggered photoelectrons transfer from CsPbBr_3_ to active sites in GDY (Fig. 13f).

#### ***Type-II Heterojunctions for Photocatalytic CO***_***2***_*** Reduction***

Type-II heterojunction with efficient spatial separation of charge carriers has been constructed to enhance the performance of CO_2_ reduction. Transition-metal oxides, chalcogenides, and sulfide, such as Co_3_O_4_ [122], TiO_x_ [123], ZnO [124], CdSe [125], etc., have shown great promise to form type-II heterojunction with MHPs. For instance, CsPbBr_3_ NCs loaded on a hierarchical branched ZnO nanowire/microporous graphene scaffold were reported [124], achieving a boosted photocatalytic performance with a photoelectron consumption rate of 52.02 μmol g^−1^ h^−1^. Gong et al*.* synthesized a zero-dimensional CsPbBr_3_/CdSe heterojunction through a thermal injection method [125]. DFT calculations indicated that the strong interactions of Pb-Se and Br-Cd chemical bonding in the type-II heterojunction effectively facilitated electron transfer. Cs_2_SnI_6_ perovskite nanocrystal/SnS_2_ nanosheet heterojunctions with an atomic-level close-contact interface prolonged the lifetime of photogenerated electrons in SnS_2_ from 1290 to 3080 ps [21]. Other type-II heterojunctions such as CsSnCl_3_/ZnSe [16] and CsPbBr_3_/MoS_2_ [103] were also reported for photocatalytic CO_2_ reduction.

**Fig. 14:**
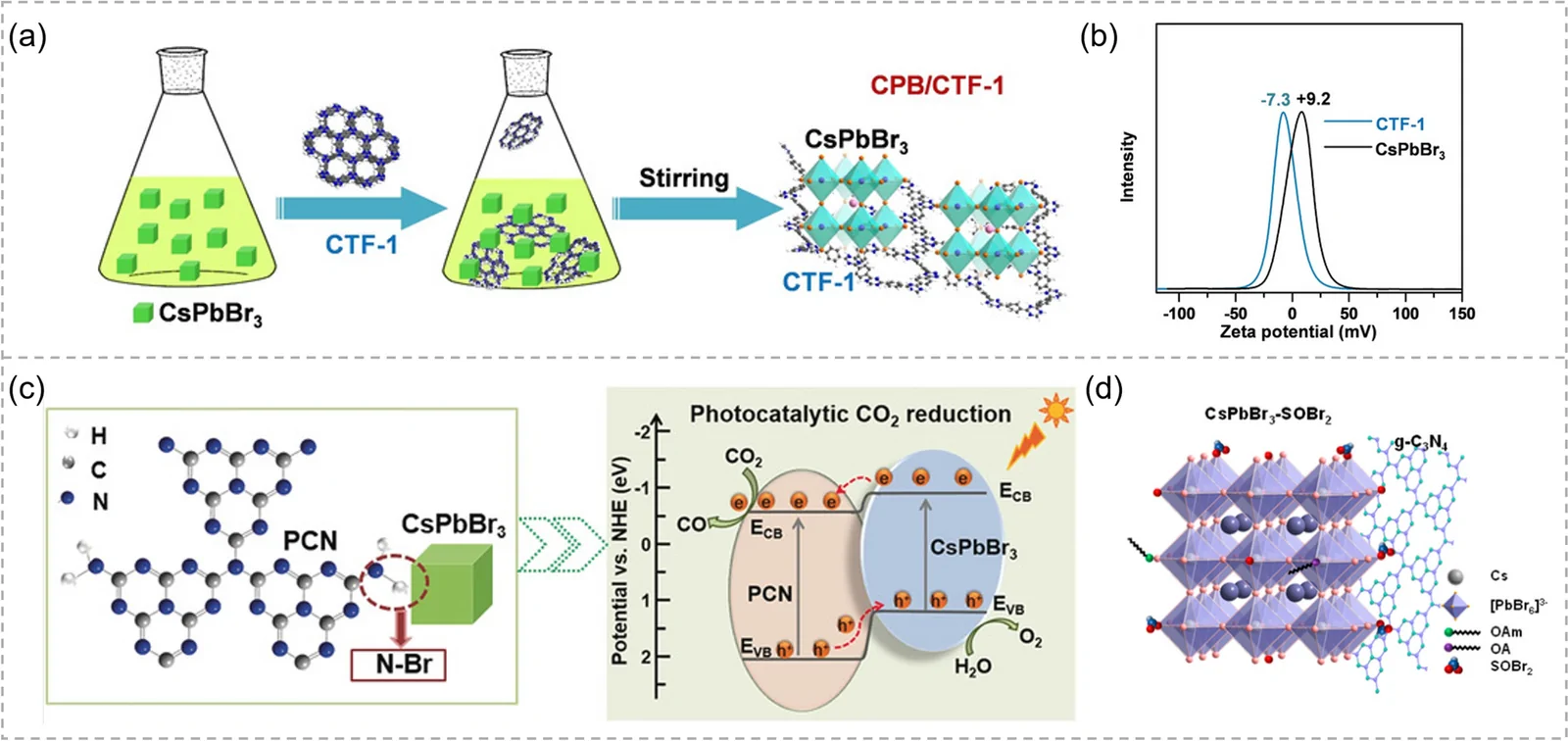
**a** Schematic illustration for the formation of the CPB/CTF-1 and **b** ξ potentials of the pristine CTF-1 and CsPbBr_3_ QDs. Reproduced with permission [61]. Copyright 2021, Wiley–VCH. **c** Illustrations of constructed CPB-PCN via N-Br bond and band alignment of the composite. Reproduced with permission [106]. Copyright 2018, Wiley–VCH. **d** Schematic illustration of the photocatalytic mechanisms of the CsPbBr_3_-SOBr_2_/g-C_3_N_4_. Reproduced with permission [126]. Copyright 2022, American Chemistry Society

Besides, metal-free organic materials have been investigated to construct type-II heterojunctions. For example, Xu et al. encapsulated CsPbBr_3_ QDs into a poly(3-hexylthiophene-2,5-diyl) (P3HT) protective layer to fabricate an efficient P3HT/CsPbBr_3_ type-II heterojunction [55]. The introduced P3HT with high carrier mobility could transport the carriers to surface quickly and act as an electron donor to donate photogenerated electrons to MHPs for CO_2_ reduction to CO and CH_4_. CTFs (covalent triazine frameworks) were coupled with CsPbBr_3_ QDs via an electrostatic self-assembly approach (Fig. 14a, b) [61]. The synergistic interactions between CsPbBr_3_ and CTF, as well as effective visible light harvesting and abundant catalytic sites, led to excellent photocatalytic activity toward CO_2_ reduction with an AQE of 0.07% at 450 nm. The protective effect of CTFs onto CsPbBr_3_ QDs enabled stable photocatalytic activity. Porous g-C_3_N_4_ with abundant amino sites on the edges were coupled with CsPbBr_3_ QDs (Fig. 14c) with the formation of N-Br bonding [106]. DFT calculations suggested the unique N-Br bonding promoted the charge carriers’ dynamics, thus leading to 15 times higher activity for CO_2_ reduction to CO compared to pure QDs. The robust connection was also established between CsPbBr_3_ medicated with SOBr_2_ and g-C_3_N_4_ (Fig. 14d) [126].

#### ***Z-Scheme Heterojunctions for Photocatalytic CO***_***2***_*** Reduction***

**Fig. 15:**
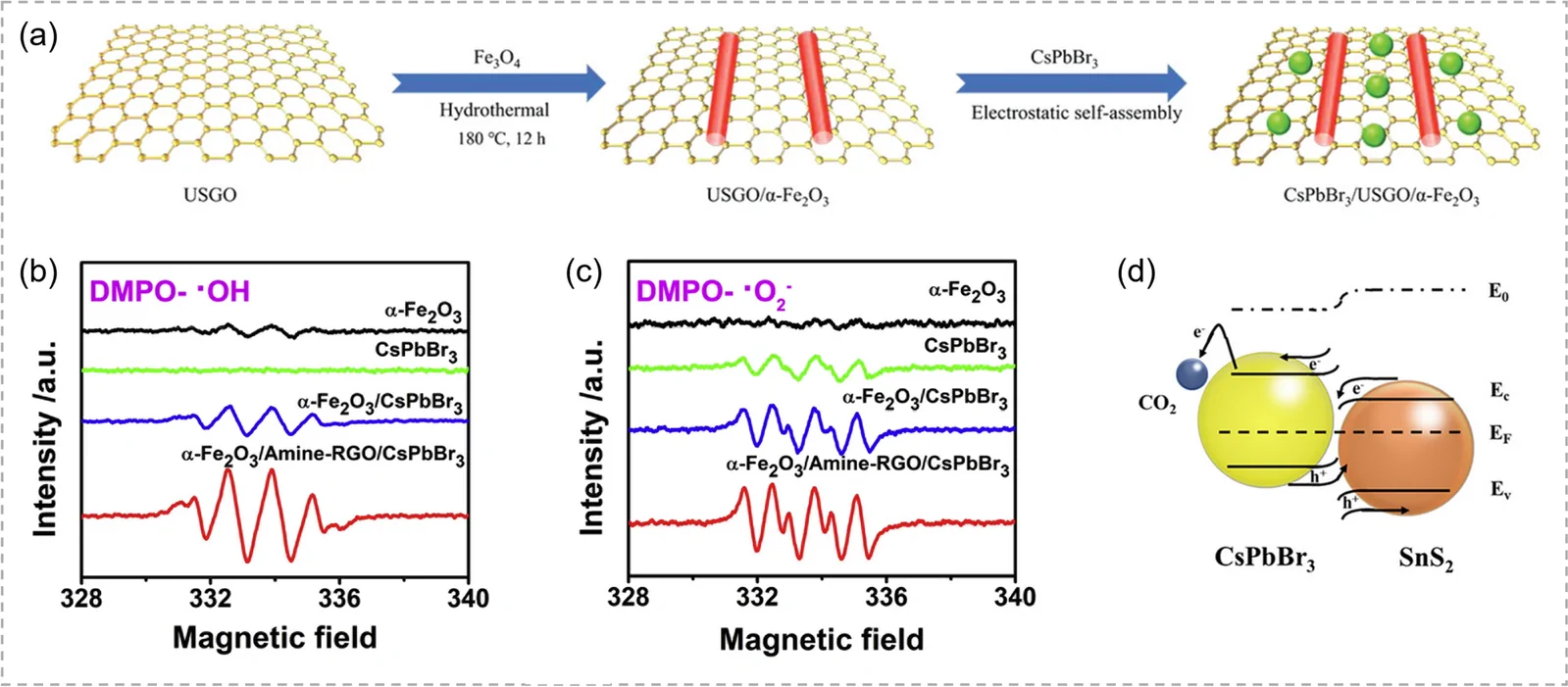
**a** Schematic illustrations of CsPbBr_3_/USGO/α-Fe_2_O_3_ synthesis processes. Reproduced with permission [72]. Copyright 2020, Wiley–VCH. EPR spectra of **b** DMPO-•OH in water/methyl cyanide and **c** DMPO-⋅O_2_^−^ in O_2_/methylcyanide in the presence of α-Fe_2_O_3_, CsPbBr_3_, α-Fe_2_O_3_/CsPbBr_3_, and α-Fe_2_O_3_/amine-RGO/CsPbBr_3_. Reproduced with permission [107]. Copyright 2020, Elsevier. **d** Mechanism of charge-carrier transfer process of CO_2_ reduction based on SnS_2_/CsPbBr_3_ NC heterojunction. Reproduced with permission [109]. Copyright 2022, Wiley–VCH

The mostly reported materials for MHPs-based Z-scheme heterojunctions were transition-metal oxides [72, 107, 108, 127, 128] and sulfide [109]. An all-solid-state Z-scheme system was fabricated with CsPbBr_3_ and α-Fe_2_O_3_ using ultrathin and small-size graphene oxide (USGO) nanosheets as the electron mediator [72]. CsPbBr_3_ and α-Fe_2_O_3_ can be closely anchored onto USGO nanosheets through chemical bonds with abundant function groups on USGO surface (Fig. 15a), i.e., carboxyl, hydroxyl, thus facilitating charge separation and transfer (Fig. 15b, c) [107]. A high electron consumption rate of 147.6 μmol g^−1^ h^−1^ for CO_2_-to-CO conversion was achieved. SnS_2_ without catalytic activity toward CO_2_ reduction was deposited on CsPbBr_3_ NCs to form SnS_2_/CsPbBr_3_ Z-scheme heterojunction, and 2.4-fold higher activity of CO was obtained (Fig. 15d) [109]. Inorganic oxide perovskite with the formula of ABO_3_ (where A is an alkaline-earth metal; B is transition metal) was also developed. Li et al. reported one example of direct Z-scheme heterostructure Cs_2_AgBiBr_6_/Sr_2_FeNbO_6_ (CABB/SFNO) double perovskites [129]. The different work functions and Fermi levels enabled the electron transfer from the CB of SFNO to VB of CABB under light irradiation, illustrating a direct Z-scheme electron transfer mode. Fu et al*.* have fabricated a Cu-BTC-encapsulated CsPbBr_3_ QDs core/shell Z-scheme heterojunction. The existing coating layer improved the stability in moisture remarkably [130].

Metal-free carbon nitride materials were also introduced to fabricate Z-scheme heterojunction for CO_2_ reduction. Cs_2_AgBiBr_6_@g-C_3_N_4_ Z-scheme was constructed by regulating the used solvent during in situ assembly process [49]. By combining the reduction ability of CB of g-C_3_N_4_ and oxidization ability of VB of Cs_2_AgBiBr_6_, Z-scheme system showed a high CH_4_ selectivity in CO_2_ photoreduction. P-modified g-C_3_N_4_ was further incorporated to fabricate P-CN/CsPbBr_3_ [131]. Because the CB position is not negative enough to reduce CO_2_ to CO, the Z-scheme mechanism was considered to be formed instead of type-II. The P-CN/CsPbBr_3_ obtained a superior CO_2_ reduction to CO with the yield enhancement of 7.2- and 1.2-fold compared to pristine P-CN and CsPbBr_3_, respectively.

#### ***S-Scheme Heterojunctions for Photocatalytic CO***_***2***_*** Reduction***

Yu et al. reported the first work about metal oxide/MHPs (TiO_2_/CsPbBr_3_) S-scheme heterojunction for photocatalytic CO_2_ reduction [132]. DFT calculations revealed that the internal electric field promoted the transfer of photogenerated electrons in CB of TiO_2_ to VB of CsPbBr_3_. An et al. modulated the exposed facets of TiO_2_ in S-scheme heterojunction to simultaneously optimize the interfacial and surface electronic structures for directional charge migration [133]. Other metal oxides including WO_3_ [134, 135], ZnO [136], SnO_2_ [116, 137], and Cu_2_O [138] were also studied to construct S-scheme heterojunctions. Lu et al*.* deposited lead-free Cs_3_Sb_2_Br_9_ nanocrystals onto the surface of Co_3_O_4_ to generate a S-scheme heterojunction via polymer-assisted growth method. Due to the enhanced charge separation efficiency, the composites showed excellent activity of CO_2_ reduction to CO with the yield of 700.7 μmol g^−1^ h^−1^ [139].

Bismuth-based materials with highly anisotropic Fermi surface, small electron effective mass and band gap, and large electron meant free path have attracted great interests to construct S-scheme heterojunctions [140–142]. A bismuthine/CsPbBr_3_ QDs S-scheme heterojunction was prepared through in situ growth [143]. The introduction of narrow band gap bismuthine enhanced light absorption abilities and suppressed the recombination of charge carriers. BiOCl with a band gap of 3.2–3.6 eV was used to fabricate 2D/2D CsPbBr_3_/BiOCl S-scheme photocatalyst, and a much enhanced CO_2_ photoreduction performance was obtained [60]. Other Bi-based materials, such as 2D Bi_2_WO_6_ (BWO) [144, 145], Bi-MOF [114], and BiVO_4_ [53], were also reported. In addition, lead-free Cs_2_AgBiBr_6_/BiVO_4_ S-scheme heterojunction was prepared through electrostatic assembly. Under the optimal conditions, the composite showed 9.2-time enhancement compared to Cs_2_AgBiBr_6_ alone. DFT calculations revealed that the main active sites for CO_2_ photocatalytic reduction were Ag sites [146].

Except for metal materials, g-C_3_N_4_ was designed to encapsulate CsPbBr_3_ nanoparticles to obtain a water-stable core/shell S-scheme heterojunction (m-CN@CsPbBr_3_) [147]. The rich adsorption and activation sites of CO_2_ molecules as well as polar surface resulted in an outstanding activity of CO_2_-to-CO with the yield of 42.8 μmol g^−1^ h^−1^. S-doped g-C_3_N_4_ was further used to construct S-g-C_3_N_4_/CsPbBr_3_ S-scheme heterojunction, which not only improved the charge separation, but also enhanced the visible-light response.

#### ***Hybrid Heterojunctions for Photocatalytic CO***_***2***_*** Reduction***

Besides the above single heterojunctions, hybrid heterojunctions, consisting of two or more heterojunctions, have also been constructed. For example, Lu et al*.* have developed a ternary MHPs-based heterojunction, TiO_2_/MHP/GDY, in which a Z-scheme and a typical type-I heterojunction were formed, through a self-templating method combined with sequential deposition technology [148]. Because of the unique sandwich structure, the photocatalysts possessed an exceptional stability in water-containing environments for durations exceeding 200 h. Hou et al*.* reported a novel composite photocatalyst system for artificial photosynthesis through embedding MHPs in functionalized MOF glass, in which a type-I and type-II heterojunction interfaces were simultaneously formed. Photoinduced electrons were efficiently generated under light irradiation and transferred for coenzyme regeneration, which could then be consumed by immobilized enzymes for CO_2_ reduction to formic acid [149].

### MHPs-Based Heterojunctions for Photocatalytic Organic Pollutant Degradation

With the rapid industrialization and urbanization, water pollution has become a global issue. The most representative pollutants are toxic organic dyes, such as rhodamine B (RhB), 4-nitrophenol (4-NP), and methylene blue (MB). Photocatalytic technology is an efficient and cost-effective way to degrade the pollutant into low toxicity inorganic small molecules. For photo-degradation organic pollutants, photo-induced electrons in CB of semiconductors firstly reduce O_2_ to generate superoxide radicals ^•^O_2_^−^ and then react with target pollutants. The degradation efficiency is denoted as C/C_0_, where C_0_ and C represent the absorption intensity of dye before and after illumination. Most MHPs possess high CB levels, which are feasible enough to produce reactive oxygen species. Several results about MHPs degrading organic dyes have been reported, especially MHPs-based heterojunctions [150].

#### Schottky Junctions for Photocatalytic Organic Pollutant Degradation

**Fig. 16:**
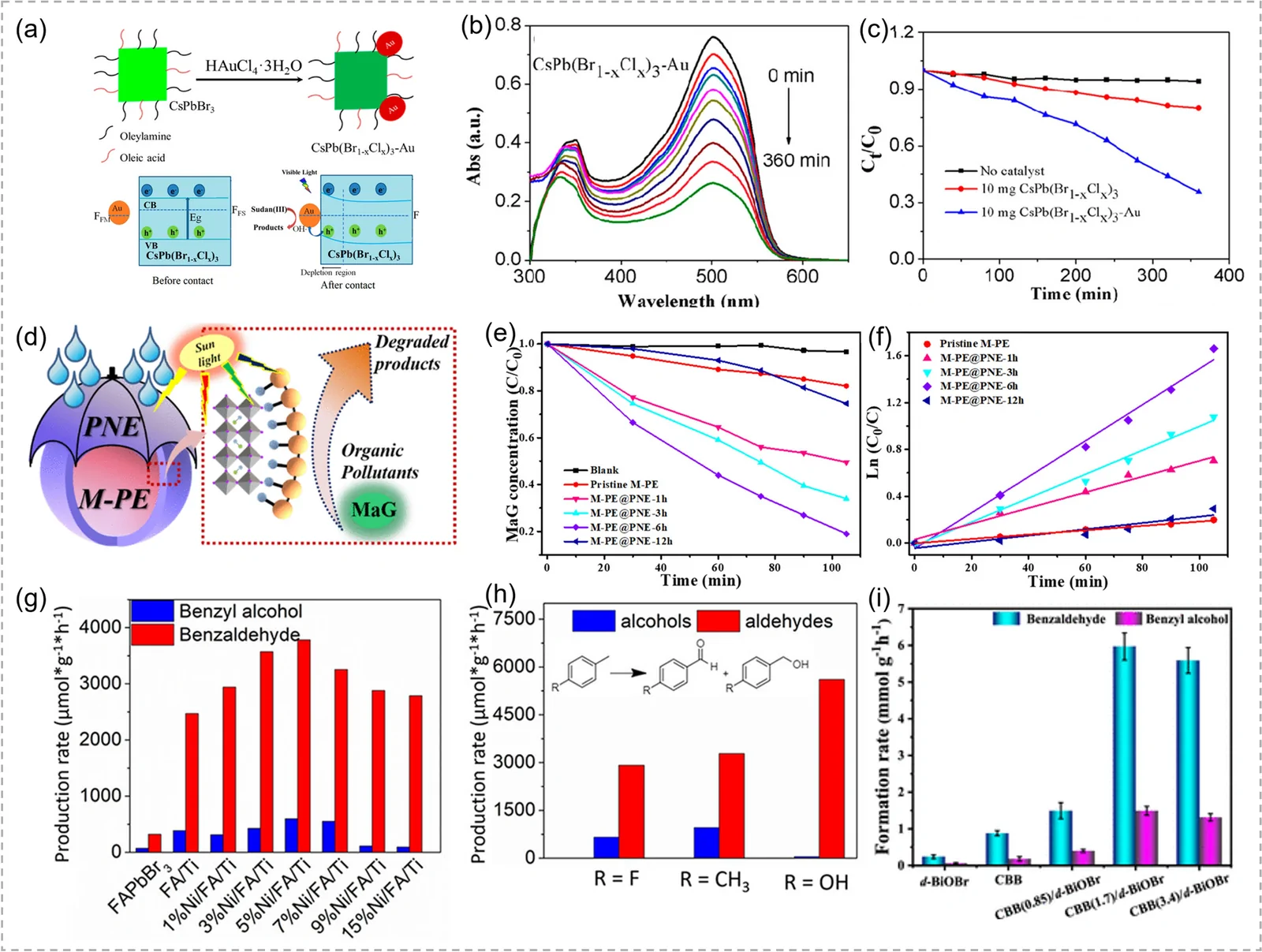
**a** Reduction of Au(III) at the surface of CsPbBr_3_ NCs to form CsPb(Br_1-x_Cl_x_)_3_-Au heterojunctions and proposed photocatalytic mechanisms, **b** absorption spectra of Sudan Red III degraded using CsPb(Br_1-x_Cl_x_)_3_-Au heterojunctions, and **c** concentration (C_t_/C_0_) changes *vs* time of Sudan Red III using different catalysts. Reproduced with permission [151]. Copyright 2019, American Chemistry Society. **d** Schematic illustration of M-PE@PNE photodegradation organic pollutants, **e** plots of C/C_0_ *vs* time for the photocatalytic degradation of MaG (3.0 mg L^−1^) in the presence of pristine M-PE and MPE@PNE-1/3/6/12 h under visible light, and **f** plots of ln(C_0_/C) *vs* irradiation time with the fitting results included. Reproduced with permission [155]. Copyright 2020, American Chemistry Society. Photocatalytic oxidation of C(sp^3^)-H in **g** Tol and **h** substituted toluene over NiO_x_/FAPbBr_3_/TiO_2_. Reproduced with permission [157]. Copyright 2019, American Chemistry Society. **i** Comparison of photocatalytic toluene oxidation over d-BiOBr, CBB, and the CBB/d-BiOBr composite. Reproduced with permission [158]. Copyright 2022, American Chemistry Society

In 2019, Huang et al*.* deposited Au nanoparticles on CsPb(Br_1-x_Cl_x_)_3_ to degrade Sudan Red III under visible light irradiation (Fig. 16a) [151]. The composite achieved 71% of Sudan Red III degradation within 6 h (Fig. 16b, c). To avoid the pollution from MHPs, Cs_2_AgBiBr_6_ was employed to fabricate Pt/Cs_2_AgBiBr_6_ for degradation [152], in which four different dyes including Rhodamine B (RhB), Rhodamine 110 (Rh110), Methyl red (MR), and Methyl orange (MO) were involved in the investigation. In addition, γ-CsPbI_3_ NCs/WS_2_ Schottky junction was also reported to degrade Methylene blue [35]. The superior carrier-transport property of few-layered WS_2_ nanosheets enabled a high photocatalytic degradation efficiency of nearly 100% in 30 min.

#### Type-I Heterojunctions for Photocatalytic Organic Pollutant Degradation

Liu et al*.* reported one type-I heterostructure example of Ag-CsPbBr_3_/CN toward 7-aminocephalosporanic acid (7-ACA) degradation [153]. A superior photocatalytic activity was achieved with degradation efficiency of 92.79% toward 7-ACA within 140 min. Experiment results revealed that the improved photocatalytic activity was attributed to the excellent adsorption capacity, enhanced light harvesting, and reduced charge recombination. Photoinduced holes and hydroxyl radicals played major roles, which could attack 7-ACA molecules to degrade into CO_2_, H_2_O and other small molecules, while electrons and superoxide radical played minor roles based on relative reactive-species-trapping experiments.

#### Type-II Heterojunctions for Photocatalytic Organic Pollutant Degradation

Owing to the facilitated charge-carrier dynamics, Chattopadhyay et al*.* embraced polymeric graphitic carbon nitride (g-C_3_N_4_) and all-inorganic cesium lead halide perovskite (CsPbBrCl_2_) to construct type-II heterojunction [154]. The generation of active radicals resulted in much improved performance in cationic and anionic dyes degradation. In addition, poly(norepinephrine) (PNE) was used to encapsulate MHPs to improve chemical stability. The existing hydroxyl group in the side chain results in a well-controlled, ultrasmooth coating layer and reduced aggregation during polymerization. MAPbBr_3_ (M-PE) NPs were covered with PNE layer via a surface growth method [155]. Benefitting from the coating layer of PNE, the obtained M-PE@PNE core@shell not only showed great stability but also exhibited excellent malachite green degradation efficiency (81% in less than 2 h), almost 8-time enhancement compared with pristine M-PE NPs (Fig. 16d-f). Abdukayum et al*.* reported a lead-free MHPs-based heterojunction photocatalyst of Cs_2_AgBiI_6_/g-C_3_N_4_ for RhB degradation, outperforming Cs_2_AgBiI_6_ by a factor of 1.3. The improved photocatalytic performance was attributed to the improved charge separation and transfer across the heterojunction interface owing to the band alignment effects [156].

#### Z-Scheme Heterojunctions for Photocatalytic Organic Pollutant Degradation

In 2018, Zeng et al*.* prepared Z-scheme MASnI_3_/TiO_2_ heterojunctions via a hydrothermal method [159]. The calcined MASnI_3_/TiO_2_ (1:6) enabled RhB concentration decreased to 1% within 50 min of light irradiation. Cs_3_Bi_2_I_9_/g-C_3_N_4_ constructed based on nitrogen–iodine chemical bonding was also reported as a new binary photocatalyst for degradation of RhB, MB, MO and the mixture of MB and MO [160]. A ternary Z-scheme Ag/CsPbBr_3_/Bi_2_WO_6_ photocatalyst was reported by Xu et al., in which Ag nanoparticles were used as charge mediators in heterojunction [161].

#### S-Scheme Heterojunctions for Photocatalytic Organic Pollutant Degradation

S-scheme CsPbBr_3_-rGO/Bi_2_WO_6_ heterojunction was synthesized via electrostatic adsorption by forming -COO-Bi-O- bond, in which mercaptopropionic acid (MPA) was capped onto CsPbBr_3_ surface [162]. The concentrations of norfloxacin were tested by time-dependent UV spectrum and time-dependent HPLC spectrum. The results revealed that the degradation rate was about 66.79% with 120 min under visible light irradiation. The excellent performance was owing to expanding light-harvesting scope, enhanced charge-carrier transport property, and exposed active species.

### MHPs-Based Heterojunctions for Photocatalytic Organic Synthesis

Value-added chemicals, pharmaceuticals, and preservatives synthesized by organic transformation from raw materials via heterogeneous photocatalytic technique have attracted wide interests in recent years. According to the kinds of substrate molecules, we have classified organic synthesis reaction into C(sp^3^)-H bond activation, radical addition, thiols oxidation, and alcohols oxidation.

#### ***MHPs-Based Heterojunctions for C(sp***^***3***^***)-H Bond Activation***

The activation of inert C(sp^3^)-H bond is one of the most challenging reactions in organic synthesis. Traditional C(sp^3^)-H bond activation is achieved by thermal catalysis with the help of noble metals under high temperature and pressure. Recently, heterogeneous photocatalysis with MHPs as photocatalysts to activate C(sp^3^)-H bond using oxygen as oxidant seems to be a potential avenue.

*Type-II heterojunctions for C(sp*^*3*^*)-H bond activation:* Li et al*.* fabricated a series of CsPbBr_3_/TiO_2_ type-II heterojunction photocatalysts via the ligand-assisted reprecipitation method toward aromatic C(sp^3^)-H bond conversion with toluene as substrate [163]. The optimal sample exhibited a fourfold and threefold higher activity than that of bare CsPbBr_3_ and TiO_2_, respectively. In addition, TiO_2_ and NiO_x_ as electron and hole transporting layers were introduced, respectively, to form NiO_x_/FAPbBr_3_/TiO_2_ to promote charge separation and transfer, in which two type-II heterojunctions were formed [157]. An external quantum efficiency of 0.25% at 400 nm was achieved (Fig. 16g, h). To make reaction conditions greener, lead-free MHPs were involved in designing Cs_3_Bi_2_Br_9_/CdS [45] and Cs_3_Bi_2_Br_9_/g-C_3_N_4_ [164] heterojunctions for C(sp^3^)-H bond activation.

*Z-scheme heterojunctions for C(sp*^*3*^*)-H bond activation:* A Z-scheme structure modulated by interfacial chemical bonding through in situ growth of Cs_3_Bi_2_Br_9_ nanodots on defective BiOBr nanosheets for toluene oxidation was constructed (Fig. 16i) [158]. Owing to the formed interfacial internal electric field, Bi-Br bond acted as a direct avenue for electron transfer, leading to a higher charge localization on the surface. In situ infrared Fourier transform spectroscopy and DFT calculations revealed that surface localization of holes was essential for toluene adsorption and the dissociation of C(sp^3^)-H and identified the key intermediates and active sites. Chen et al. designed Co_x_Bi_2-x_O_2_CO_3_ nanosheets and used as self-template to epitaxially grow Cs_3_Bi_2_Br_9_ via Bi atom bridge [165]. The incorporated Co^3+^ played critical roles in heterojunction growth by regulating electronic structure and growth dynamics of Cs_3_Bi_2_Br_9_, enabling significantly enhanced toluene photo-oxidation performances.

#### MHPs-Based Heterojunctions for Radical Addition

Recently, Ravelli et al*.* reported one example of MHPs-based heterojunctions for radical addition reaction. Lead-free perovskite, Cs_2_AgBiBr_6_, was selected to couple with g-C_3_N_4_ to construct a type-II heterojunction [166]. The improved charge-carrier dynamics enabled the atom transfer radical addition-type carbohalogenation of multiple C–C bonds, such as alkenes, alkynes, with alkyl halides.

#### MHPs-Based Heterojunctions for Thiols Oxidation

Disulfides are of interest as protecting groups in synthetic applications and as vulcanizing agents for rubber, which were usually obtained from oxidative coupling of thiols. To avoid overoxidation, various stoichiometric oxidants were used. In 2025, Le et al*.* reported a Schottky junction of CsPbBr_3_/Ti_3_C_2_T_x_ MXene for thiols oxidation. The production rate was up to 29,700 μmol g^−1^ h^−1^ in air without any additional oxidants [167].

#### MHPs-Based Heterojunctions for Alcohols Oxidation

The selective oxidation of alcohols into carbonyls is an important reaction in organic synthesis, which is usually performed by stoichiometric inorganic/organic oxidants, such as permanganate, dichromate, 2,2,6,6-tetramethylpiperidine oxide, and noble metal catalysts. To avoid these harsh conditions, photocatalytic techniques have been employed to oxide alcohol using O_2_ as sole oxidant, resulting in high conversion and selectivity.

*Type-II heterojunctions for alcohols oxidation:* In 2018, Roeffaers et al. built a type-II heterojunction of FAPbBr_3_/TiO_2_ with antisolvent precipitation method for photocatalytic oxidation of benzyl alcohols in a polar solvent [168]. The highest photocatalytic conversion of benzyl alcohol peaked at 63%, totally a 4-time enhancement over the pure controls. Moreover, the composite CsPbX_3_/W_18_O_49_ showed high selectivity toward selective oxidation of benzyl alcohol to benzaldehyde and a conversion up to 72%, which is 11, 10, and 2.5 times higher than that of pure CsPbCl_3_, CsPbBr_3_, and W_18_O_49_, respectively [68].

*Z-scheme heterojunctions for alcohols oxidation:* Zhang et al. constructed Z-scheme heterojunction FAPbBr_3_ nanocrystals/WO_3_ with an outstanding permanence of benzyl alcohol oxidation to benzaldehyde with selectivity of 99% [169]. Astonishingly, after the size of FAPbBr_3_ reduced to quantum dots, benzoic acid was found as the main product with 90% selectivity. FAPbBr_3_/Bi_2_WO_6_ was developed for benzyl alcohol oxidation to benzaldehyde coupled with CO_2_ reduction to CO [63]. Ultrafast transient infrared absorption revealed photocarrier dynamics and demonstrated Z-scheme charge transfer mechanisms.

## Conclusions and Outlook

MHPs with high molar extinction coefficient, tunable band gap, and high carrier mobilities have aroused huge interest in photocatalytic redox reactions but still suffer from some intrinsic limitations, e.g., inferior stability, severe charge-carrier recombination, and limited active sites. Construction of heterojunctions has been demonstrated to effectively overcome these shortcomings and has made great progress. This review summarized the recent progress of MHPs-based heterojunctions (e.g., Schottky junction, type-I, type-II, Z-scheme, S-scheme) for solar-light-driven redox reactions, including H_2_ evolution, CO_2_ reduction, organic pollutant degradation, and organic synthesis. On the one hand, the formation of heterojunctions promotes spatial separation of electrons and holes, thus significantly enhancing the photocatalytic activity. On the other hand, the addition of other materials could passivate the surface of MHPs, leading to improved stability. Although MHPs-based heterojunction photocatalysts have been widely investigated and significant achievements have been made, the research of MHPs-based heterojunctions in photocatalysis is still at the preliminary stage. Several challenging issues need to be solved in the future.

I. The stability of heterojunctions needed to be improved, especially in water/aqueous solution due to the inherent nature of ionic semiconductors. Several stabilization strategies such as encapsulation, surface passivation, or compositional engineering can be considered in the future. For example, protective matrices such as porous metal–organic frameworks (MOFs), graphene oxide, or polymers (e.g., poly(norepinephrine)) can physically shield MHPs from environmental degradation while maintaining charge transport. Ligand engineering (e.g., halogen-rich surfaces or organic capping agents), which mitigates surface defects and ion migration, could improve the durability due to passivated interfacial traps.
II. The influence of heterointerface structure, such as interface locations, sizes, and coupling forces (chemical bonding or van der Waals’ interaction) on photogenerated charge-carrier dynamics and photocatalytic performance, needs to be disclosed. A more in-depth understanding of these factors would provide guidance for researchers to construct high-performance heterojunctions.
III. Advanced characterization techniques need to be involved. Although some advanced characterizations such as EPR and KPFM are utilized, they are based on the relative location of band edge and standard redox potential of active species, which are not accurate. Therefore, in situ and operando characterizations under realistic reaction conditions to reveal the fine structures and behaviors of photogenerated charge carriers during the reaction are needed. For example, in situ KPFM characterization could give information about interfacial charge redistribution under light irradiation, clarifying the role of heterojunction interfaces in charge separation. Using in situ XPS characterization to measure the surface charge transfer to reveal whether the photogenerated electrons or holes transferred to photocatalysts surface, is important for the subsequent reactions. Femtosecond time-resolved transient absorption spectroscopy could be measured to reveal the photogenerated exciton relaxation progress, which could give evidence about the interaction between exciton and reactant molecule. Moreover, operando microscopy (e.g., TEM or SEM) coupled with environmental cells could reveal the structural evolution of photocatalysts (e.g., phase stability, defect formation) during reaction, addressing stability challenges. In situ Fourier transform infrared spectroscopy (FTIR) can identify intermediate species during CO_2_ reduction or organic synthesis, linking interfacial properties to product selectivity. These techniques would bridge the gap between static characterization and real-time performance, enabling a deeper understanding of structure–activity relationships.
IV. Specific active sites need to be introduced into heterojunctions to realize target products generation. Taking CO_2_ reduction reaction as an example, improving the products selectivity is significant. In the present, the products for CO_2_ reduction were mainly simple products (e.g., CO, CH_4_) with limited selectivity for C_2_ + compounds. Introducing tailored active sites (e.g., single-atom catalysts or bimetallic cocatalysts) could steer reactions toward higher-value products like ethylene or ethanol.
V. While this review focuses on experimental advances, DFT studies have significantly contributed to understanding MHP heterojunctions. Computational works have clarified band alignment, interfacial charge transfer, and defect effects in Schottky, type-II, and S-scheme systems (e.g., CsPbBr_3_/CdSe, Cs_2_AgBiBr_6_/WO_3_). Future DFT efforts should target descriptor-based design (e.g., adsorption energies, charge localization) and multiscale modeling to bridge atomic-scale insights with macroscopic performance.
VI. The introduction of sacrificial reagents should be avoided. In most reported works, electron donors such as isopropanol, hypophosphoric acid, are involved, especially in H_2_ evolution reaction. Valuable synthesis reactions should be introduced to utilize oxidation ability of photogenerated holes or generated halogen radical anion intermediate to produce useful chemicals, which seems more promising for further applications.
VII. Most reported photocatalytic organic synthesis is only a simple oxidation reaction, such as oxidation of alcohol/aldehyde/toluene, which usually involves one substrate. More complex substrates with functional groups need to be involved. Moreover, multicomponent redox reaction should be developed to generate compounds with various functional groups as frameworks of medicine, pesticide, or maquillage, etc.

The future of MHP-based heterostructures lies in interdisciplinary efforts combining advanced characterization, computational design, and innovative reactor engineering. Addressing these challenges could unlock their full potential for sustainable solar-to-chemical conversion, impacting energy, and environmental sectors globally. Collaborative research across academia and industry will be pivotal in transitioning these materials from lab-scale breakthroughs to real-world applications.
